# Dose-response relationships of sensorimotor-based interventions on balance performance in older adults: A systematic review and meta-regression analysis

**DOI:** 10.1371/journal.pone.0354522

**Published:** 2026-07-23

**Authors:** Song Chen, Pengwei Chen, Lu Huang, Wenhao Guo, Yuanji Zhong

**Affiliations:** 1 School of Physical Education and Arts, Jiangxi University of Science and Technology, Ganzhou, Jiangxi, China; 2 School of Economics and Management, Shanghai University of Sport, Shanghai, China; 3 School of Recreational Sports and Tourism, Beijing Sport University, Beijing, China; UFPE: Universidade Federal de Pernambuco, BRAZIL

## Abstract

Falls are a severe public health challenge for older adults. Although sensorimotor-based interventions can remodel balance, their quantitative dose-response trajectories remain unknown, often leading to blindly accumulated doses. We aimed to identify optimal intervention strategies by deconstructing these non-linear dynamics. A comprehensive search of five databases up to February 2026 identified relevant randomized controlled trials (RCTs). We constructed a random-effects model using the standardized mean difference (Hedges’ g). Restricted cubic spline (RCS) and meta-regression models explored the non-linear dynamics of total intervention dose and the moderating effects of age. We included 24 studies comprising 1,110 participants. Sensorimotor-based interventions significantly improved dynamic balance (Timed Up and Go Test [TUGT]: g = −0.89, Q = 78.19; low-certainty evidence), static balance (Berg Balance Scale [BBS]: g = 0.94, Q = 21.14; high-certainty evidence), and neuromuscular control, including Center of Pressure with eyes open (COP-EO: g = −0.66, Q = 8.19; low-certainty evidence) and Center of Pressure with eyes closed (COP-EC: g = −0.34, Q = 3.54; moderate-certainty evidence). The RCS model suggested significant non-linear dose dependency for dynamic balance (P _non-linearity_ = 0.004), indicating a potential relative attenuation of effect sizes near a cumulative dose of 1000 minutes. Conversely, static balance showed no significant dose association. Increasing age significantly attenuated dynamic balance benefits (p = 0.047) but did not negatively affect static stability. The adaptive trajectories of dynamic and static balance responding to sensorimotor interventions diverge fundamentally. Based on these non-linear dose-response fluctuations and age-related characteristics, continuous time accumulation may not guarantee proportional linear returns. Optimizing fall prevention requires shifting toward precise interventions that consider dose-efficiency and age stratification rather than a “more is better” approach. **Systematic Review Registration:** INPLASY202630061.

## Introduction

As the global demographic structure irreversibly shifts toward an aging society, falls and their associated complications have emerged as a severe public health challenge that threatens the quality of life and life expectancy of older adults [1–3]. Representing the most critical modifiable risk factor for falls, the progressive decline in balance performance stems not merely from age-related musculoskeletal deterioration; it fundamentally reflects the functional degradation of the central nervous system regarding multisensory integration and motor strategy execution [4–6]. Consequently, interventions designed to reverse this neuromuscular decline have become a focal point in geriatric rehabilitation medicine. This is particularly true for sensorimotor-based interventions, which emphasize the coupling of proprioceptive input and motor output and encompass core paradigms such as sensorimotor training (ST), proprioceptive training (PT), and proprioceptive neuromuscular facilitation (PNF) [7–11].

Previous evidence-based medicine indicates that these sensorimotor-based interventions significantly optimize postural stability among older adults in complex environments by inducing neuroplastic changes within cortical and subcortical centers [12,13]. By simulating unstable sensory conditions, this intervention paradigm compels the nervous system to recalibrate the weight distribution across visual, vestibular, and proprioceptive inputs through sensory reweighting, thereby reconstructing more efficient fall-prevention mechanisms along neural pathways [14–16]. However, while the general efficacy of these paradigms is widely acknowledged, substantial research gaps persist regarding their clinical translation, specifically concerning the determination of quantitative parameters for exercise prescriptions.

Recent evidence has strengthened the clinical rationale for sensorimotor and proprioceptive exercise in older adults. A recent systematic review with meta-analysis showed that these programs can improve balance performance in aging populations, while also noting marked inconsistency in how sensorimotor and proprioceptive interventions are defined, structured, and delivered across studies [17]. Recent meta-analytic evidence further indicates that exercise programs targeting balance, strength, mobility, and sensorimotor control can reduce fall risk and improve functional balance outcomes in older adults, but these syntheses have mainly evaluated overall efficacy or broad prescription variables rather than the non-linear cumulative dose-response trajectory of sensorimotor-based interventions [18–20]. Collectively, these findings indicate that the field has moved beyond asking whether sensorimotor-based interventions are beneficial, but still lacks evidence on how cumulative exposure shapes adaptation, when benefits begin to plateau, and whether dynamic balance, static balance, and postural-control outcomes follow distinct dose-response trajectories.

Current mainstream guidelines often emphasize qualitative descriptions of training modalities when recommending intervention protocols. In contrast, the definitions of key quantitative parameters—such as frequency, single-session duration, and total intervention duration—remain overly broad and lack consideration for heterogeneity [21,22]. More importantly, previous meta-analyses have predominantly relied on linear regression models to explore the association between dose and efficacy. This statistical approach assumes a “higher dose, greater benefit” paradigm, which overlooks the non-linear characteristics inherent in the long-term adaptation processes of biological systems, including neural adaptation thresholds, diminishing marginal returns, and the physiological saturation plateau [23]. Neglecting these non-linear dynamics risks prescribing insufficient doses that fail to trigger effective adaptation, or conversely, accumulating excessive doses that result in inefficient redundancy and neural fatigue, ultimately impeding the advancement of precision rehabilitation.

Given these limitations, the present study aims to move beyond traditional linear analyses by employing a RCS model to conduct an in-depth non-linear deconstruction of the dose-response relationship between sensorimotor-based interventions and improvements in dynamic balance and static balance among older adults [24,25]. This research addresses a core question in translational medicine: is there a cumulative dose range that produces the greatest neuromuscular adaptation, and does additional training beyond this range lead to diminishing incremental benefits? Concurrently, this study quantifies the moderating effects of key demographic characteristics, such as age, on the dose-response relationship. Ultimately, these findings seek to provide robust evidence to inform the development of physiologically threshold-based, stratified, and precise exercise prescriptions for fall prevention in older populations.

## Materials and methods

This systematic review and meta-analysis strictly adhered to the Preferred Reporting Items for Systematic Reviews and Meta-Analyses (PRISMA) guidelines [26] and was registered with the International Platform of Registered Systematic Review and Meta-analysis Protocols (INPLASY) (registration number: INPLASY202630061). To align with the principles of open science and enhance the transparency and reproducibility of this research, all relevant datasets and analytical codes have been provided in the Supporting Information files.

### Databases and search strategies

A comprehensive, independent literature search was executed by two investigators (S.C. and P.C.) across five electronic databases: PubMed, EBSCOhost, Embase, the Cochrane Library, and Web of Science. The search timeframe spanned from database inception to February 5, 2026. The search strategy combined Medical Subject Headings (MeSH) and free-text terms, incorporating keywords such as Aged, Sensorimotor Training, Proprioceptive Training, Proprioceptive Neuromuscular Facilitation, and Postural Balance (Table A1 in S1 Appendix). Search syntaxes were appropriately adapted to accommodate the specific indexing systems of each database.

### Eligibility criteria

#### Inclusion criteria.

Inclusion criteria were formulated based on the PICOS framework, encompassing five dimensions: population, intervention, comparison, outcomes, and study design:

Population (P): Participants aged 55 years and older (or with a mean age ≥ 55 years) were included. Although the traditional definition of the older population typically specifies individuals over 65, current epidemiological and sports science evidence indicates that the physiological decline of human postural control often begins around the age of 50 [27,28]. Consequently, this study adopted a broader age threshold to capture the entire continuum from the early decline of balance function to advanced aging. The included population comprised healthy community-dwelling middle-aged and older adults, as well as individuals with stable chronic non-communicable conditions (e.g., frailty, sarcopenia, or high fall risk). When studies included mixed-age samples, they were retained only if the mean age of the sample was ≥ 55 years or if separate data for eligible older participants could be extracted. Participants were required to have sufficient functional capacity to safely engage in exercise-based interventions.

Intervention (I): The experimental group received standardized sensorimotor-based interventions, including ST, PT, or PNF. Eligible interventions were required to target postural control through proprioceptive input, balance challenge, or neuromuscular coordination.

Comparison (C): Control conditions involved no intervention, usual activities, or alternative exercise training. Control types were categorized into three groups: (1) passive control (e.g., unstructured training or no-treatment control); (2) usual care or routine exercise; and (3) active control (e.g., strength or aerobic training).

Outcome (O)s: Studies were required to report at least one objective outcome related to balance control. These included: (1) dynamic balance, such as the TUGT; (2) static balance, such as the BBS; and (3) neural adaptation and sensorimotor control metrics, namely COP sway parameters (Table 1).

**Table 1:** Functional categorization of outcome measures.

Dimension	Functional	Outcome	Neural Mechanisms
Balance Performance	Dynamic Balance	TUGT	Postural Transition
	Static Balance	BBS	Steady-state Maintenance
Neural Adaptation	Postural Control	COP-EO	Neuromuscular Synergy
	Sensory Control	COP-EC	Multisensory Re-weighting

Note: TUGT, Timed Up and Go Test; BBS, Berg Balance Scale; COP-EO, Center of Pressure with Eyes Open; COP-EC, Center of Pressure with Eyes Closed.

Study design (S): Only randomized controlled trials (RCTs) were included.

#### Exclusion criteria.

The following exclusion criteria were applied: (1) non-original research, such as review articles, dissertations, conference abstracts, or proceedings; (2) studies failing to report any objective indicators of neural adaptation or balance control, rendering the analysis incompatible with the research objectives; (3) duplicate publications or analyses (selecting the most recent or highest-quality version); (4) unavailable full texts; (5) non-English literature; (6) studies lacking mean and standard deviation data for outcome measures, where such values could neither be imputed from the text nor obtained from the authors; (7) non-randomized study designs; (8) unpublished research; and (9) exercise modalities deviating significantly from sensorimotor mechanisms, such as yoga, Tai Chi, or isolated resistance training.

### Selection process

Adhering to the PRISMA workflow, the initial screening of retrieved records was carried out by two reviewers (S.C. and P.C.). Following the removal of duplicates via EndNote X9 reference management software, the reviewers independently evaluated titles and abstracts to exclude studies that were clearly irrelevant or failed to meet the inclusion criteria. For studies demonstrating potential eligibility, full texts were retrieved for rigorous examination. This review specifically verified the completeness of training dose information and the reporting of objective metrics for balance control (TUGT, BBS) or neural adaptation (COP). Any discrepancies arising during the screening process were resolved through discussion; unresolved disagreements were arbitrated by a third researcher (Y.Z.). The final selection results, including the initial number of retrieved records, reasons for exclusion, and the final count of included studies, were delineated in a PRISMA flow diagram.

### Data extraction

Utilizing a standardized Excel extraction form, data extraction and subsequent quality assessments were independently completed by two trained researchers (S.C. and P.C.). Extracted data encompassed: (1) basic study characteristics (author, publication year, and country); (2) participant demographics (sample size, mean age, sex distribution, and health status); (3) intervention and control parameters, detailing the training modality (e.g., ST, PT, and PNF), control type (passive control, usual care, or active control), and training dose metrics (intervention duration in weeks, weekly frequency, single-session duration, and total training volume); (4) outcome measures, including balance control performance metrics (TUGT, BBS) and neural adaptation metrics (COP), capturing means, standard deviations, or data convertible to effect sizes; and (5) information pertaining to risk of bias. For training-dose extraction, total training volume was calculated as intervention duration in weeks × weekly frequency × single-session duration in minutes. When one of these three dose components was not directly reported, the full text, tables, figures, and supplementary materials were checked to determine whether the missing value could be derived. Extracted datasets were subsequently cross-verified by both researchers, with any disagreements resolved via discussion or third-party (Y.Z.) adjudication.

### Risk of bias

The methodological quality and potential risk of bias for all included RCTs were systematically appraised by two independent reviewers (S.C. and P.C.) using the Revised Cochrane Risk-of-Bias Tool for Randomized Trials (RoB 2.0). RoB 2.0 encompasses five primary domains: bias arising from the randomization process, bias due to deviations from intended interventions (effect of assignment to intervention or effect of adhering to intervention), bias due to missing outcome data, bias in measurement of the outcome, and bias in selection of the reported result [29]. The overall risk of bias for each study was synthesized based on the judgments across these five domains. A study was categorized as “low risk” if all domains were evaluated as low risk; “some concerns” if at least one domain raised some concerns without any high-risk domains; and “high risk” if any single domain was judged as high risk. Disagreements between reviewers were initially addressed through negotiated discussion, with a third reviewer (Y.Z.) intervening to reach a final decision if consensus could not be achieved.

### Certainty of evidence

Following the risk-of-bias appraisal, the certainty of evidence for each synthesized outcome was evaluated adhering to the Grading of Recommendations Assessment, Development and Evaluation (GRADE) framework [30]. Two independent investigators (S.C. and P.C.) performed this assessment, with any emerging discrepancies resolved through consensus-driven discussion or adjudicated by a third reviewer (Y.Z.). Within this paradigm, evidence derived from randomized controlled trials initially holds a high-certainty rating, which is subsequently subject to downgrading across five critical domains: risk of bias, inconsistency, indirectness, imprecision, and publication bias [31]. The identification of serious or very serious limitations within these domains warranted a reduction in the confidence level by one or two tiers, respectively. Consequently, the final evidence was stratified into four certainty levels, categorized as high, moderate, low, and very low, reflecting the degree of confidence that the estimated effect accurately mirrors the true underlying effect [32].

### Statistical analysis

All qualitative and quantitative synthesis procedures for this systematic review and meta-analysis were executed within the R computing environment (version 4.5.2; R Foundation for Statistical Computing, Vienna, Austria), primarily utilizing core packages such as meta, metafor, and dmetar to construct statistical models [33,34]. For the continuous outcomes of balance performance, the standardized mean difference (SMD) was uniformly adopted as the fundamental effect size for pooling. This approach effectively accommodates inherent variations in measurement scales across studies, particularly the methodological heterogeneity introduced by modified administration procedures for the TUGT, BBS, and COP. The SMD was subsequently converted to Hedges’ g and its 95% confidence interval (CI) to effectively correct for potential estimation bias associated with small sample sizes—a necessary adjustment given the relatively small cohorts in several included studies [35]. Between-study heterogeneity was quantitatively assessed using Cochran’s Q test and the I^2^ statistic [36]. Considering the inevitable inherent clinical heterogeneity across different sensorimotor-based interventions and participant populations, a random-effects model based on restricted maximum likelihood (REML) estimation was uniformly applied to ensure the external validity and robustness of the pooled results for all primary and secondary outcomes [37]. The overall distribution of effect sizes and their corresponding confidence intervals were visualized using high-resolution forest plots. Additionally, orchard plots were introduced to further illustrate the data dispersion and the precise positioning of the pooled effects [38,39].

#### Heterogeneity and subgroup analyses.

To trace potential drivers of heterogeneity, predefined multidimensional subgroup analyses were conducted, encompassing health condition, intervention total sessions, intervention total dose, and age categories. By estimating the pooled Hedges’ g for each stratum and applying Cochran’s Q test, the divergence of effect sizes across different clinical intervention characteristics was evaluated. Based on this, through an in-depth examination of the distribution characteristics of each outcome, high-impact studies and potential outliers that might induce non-random bias were accurately identified, providing targeted clues for subsequently elucidating the sources of heterogeneity.

#### Meta-regression and dose-response analysis.

To explore the underlying moderating mechanisms of intervention total dose and demographic covariates on the outcomes, a univariate random-effects meta-regression model (based on the REML framework) was established as the primary analytical tool [40]. For continuous baseline characteristics such as age, linear regression models were fitted to quantify their moderating direction on the net intervention effect, and bubble plots were used to visually map the linear trajectory between the covariate gradients and effect size variations. In analyzing the dose-response relationship of intervention duration, this study overcame the limitations of traditional linear extrapolation by adopting a RCS model to perform non-linear fitting on the total training dose [24,25]. This procedure involved appropriately setting knots and utilizing the Wald test to evaluate the statistical significance of non-linear associations. Accordingly, non-linear dose regression plots were generated to precisely capture the optimal dose window for inducing neural adaptation and potential inflection points of diminishing marginal returns.

#### Assessment of publication bias.

Recognizing that the reliability of evidence synthesis is highly dependent on the effective management of bias risk, this study established a multidimensional assessment framework integrating visual diagnostics and quantitative tests. Contour-enhanced funnel plots were introduced to accurately identify the causes of plot asymmetry by embedding statistical significance contours within the effect distribution space [41,42]. To circumvent the potential subjectivity of purely visual judgments, the assessment of asymmetry was further supplemented with objective quantification using Egger’s linear regression test. If the testing pipeline detected significant small-study effects or potential bias signals, the non-parametric trim-and-fill method was subsequently employed. This method estimated and iteratively imputed hypothetically missing studies, recalculated the adjusted pooled effect size, and simultaneously generated trim-and-fill funnel plots to visually display the shifts in data clustering before and after the simulated imputation [43]. Furthermore, to confirm the stability of the overall conclusions, a leave-one-out sensitivity analysis was conducted. By sequentially removing a single study and recalculating the pooled effect, the perturbing impact of extreme data or specific studies on the overall pooled estimate was strictly monitored, thereby establishing a robust dual evidence-based safeguard for the original meta-analysis conclusions [44].

## Results

### Study selection

**Fig 1:**
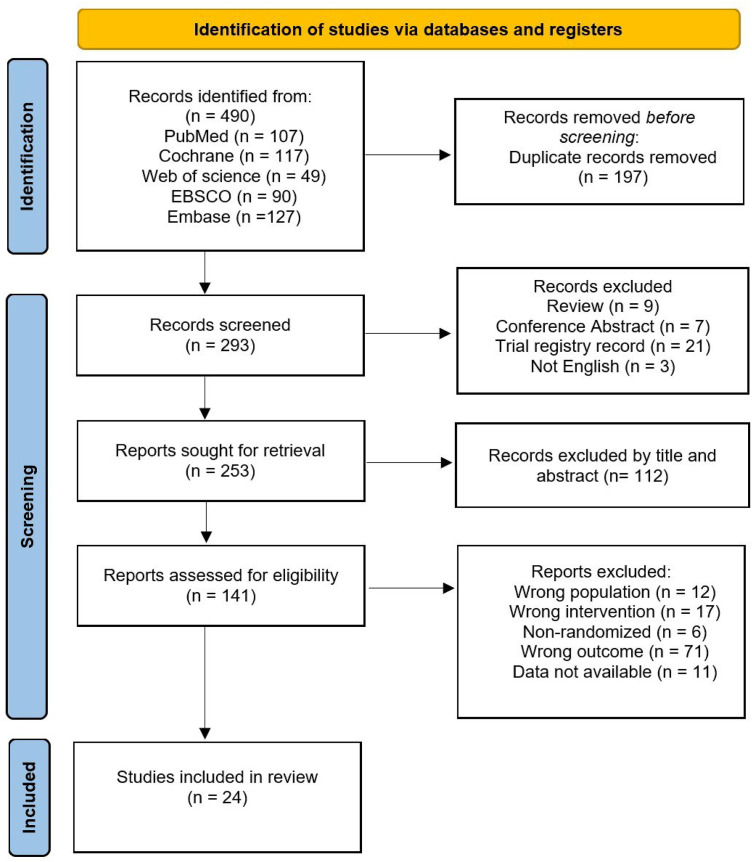
PRISMA (Preferred Reporting Items for Systematic Reviews and Meta-Analyses) flowchart identifying the study selection process.

A comprehensive literature search yielded 490 records (Fig 1). Following the predefined inclusion and exclusion criteria, two independent reviewers screened titles and abstracts, subsequently conducting full-text evaluations. Ultimately, 24 eligible RCTs were included in the final analysis [8,45–67]. The detailed screening process is depicted in the PRISMA flow diagram (Fig 1).

### Study characteristics

This systematic review and meta-regression analysis included a total of 24 studies comprising 1,110 older adult participants. The publication years ranged from 2001 to 2026, with a broad geographical distribution spanning 11 countries and regions: Brazil, Egypt, Germany, Greece, India, Iran, Italy, South Korea, Pakistan, Spain, and Turkey (Table A2 in S1 Appendix).

Participant characteristics: The age characteristics of the included studies were categorized into three tiers based on baseline mean age: < 70 years (n = 14), 70–80 years (n = 8), and >80 years (n = 2). Participants presented with various underlying conditions, including osteoporosis (n = 1), chronic diseases (n = 2), arthritis (n = 4), and diabetes (n = 4), yet all maintained the functional capacity to participate in exercise interventions. The overall sample was predominantly female, though several studies focused exclusively on male or mixed-sex cohorts.

Intervention modalities: Experimental groups received three categories of neuromuscular training modalities: ST (n = 10), PT (n = 7), and PNF (n = 7). Control conditions consisted of no intervention (n = 6), usual care or routine exercise (n = 15), and alternative active interventions such as resistance training, Swiss ball training, aerobic exercise, and dance therapy (n = 3).

Training protocol characteristics: With the exception of one study that did not explicitly report the intervention dose, the remaining studies implemented intervention durations ranging from 4 to 18 weeks, with frequencies of 1–3 sessions per week and single-session durations spanning 15–90 minutes. Generally, the majority of studies adopted moderate-intensity training protocols lasting 8–12 weeks, conducted 2–3 times weekly for 30–60 minutes per session.

Outcomes: The included studies widely utilized standardized balance assessment instruments. These encompassed dynamic balance indicators, specifically the TUGT (K = 19); static balance indicators, primarily the BBS (K = 12); and neural adaptation and sensorimotor control indicators, namely COP sway parameters (K = 7). Together, these metrics comprehensively capture the multidimensional manifestations of balance function in older adults.

### Risk of bias assessment results

**Fig 2:**
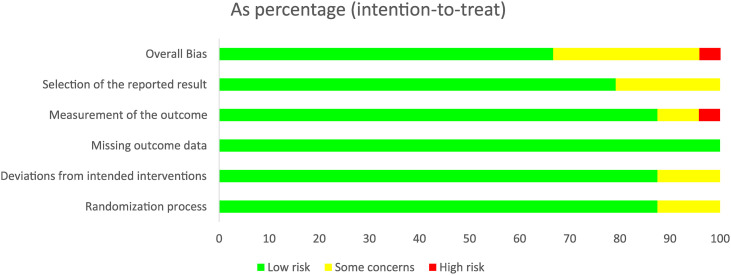
Risk of overall bias.

Fig 2 presents the overall distribution of the RoB 2.0 risk of bias assessment for the 24 included studies (detailed domain-specific assessment results for each independent study are available in S2 Appendix). Among these, 16 studies (66.7%) were rated as low risk, seven studies (29.2%) as having some concerns, and one study (4.2%) as high risk. This distribution indicates that the vast majority of studies possess robust internal validity, although a minority exhibit potential biases in methodological design and reporting. All 24 included studies utilized randomized controlled trial designs. Specifically, one study demonstrated a high risk of bias in the measurement of the outcome [51]. Three studies showed “some concerns” regarding bias arising from the randomization process [45,57,60]. Furthermore, no bias due to missing outcome data was observed across the 24 studies. Regarding bias in the selection of the reported result, five studies lacked sufficient information for accurate evaluation and were thus categorized as having “some concerns” [45,51,57,58,67]. Overall, the included studies present a low risk of bias, ensuring the robustness of the meta-regression findings in this study. Subsequent meta-regression analyses further evaluated the potential impact of “high-risk” studies on the pooled effects.

### Overall meta-analysis of balance performance

This study quantitatively synthesized the outcome measures utilizing a predefined random-effects model based on REML, visualizing the overall effects and data distribution characteristics through forest and orchard plots (Table 2). For interpretation of effect direction, lower values indicated better performance for TUGT and COP outcomes; therefore, negative Hedges’ g values reflected improvement in these outcomes. In contrast, higher values indicated better performance for BBS; therefore, positive Hedges’ g values reflected improvement in static balance. Regarding dynamic balance, 17 original articles comprising 19 independent comparisons assessing the TUGT were included (N = 889). The number of independent comparisons (K) exceeds the number of original articles because several studies employed multi-arm designs with multiple independent intervention groups [8,45,46,48,50,52,54–56,59–61,63–67]. The pooled effect size indicates that the TUGT completion time in the intervention group was significantly shorter than that in the control group (Hedges’ g = −0.89, 95% CI: −1.20, −0.57). Visual distribution characteristics from the orchard and caterpillar plots (Figs A1–A3 in S1 Appendix) reveal that, although the main intervention effect demonstrates a significant beneficial trend, the effect sizes of individual studies display a broad dispersion band across the regression line, indicating high inter-study heterogeneity (I^2^ = 79.26%, Q = 78.19, p < 0.001).

**Table 2:** Pooled effect sizes and heterogeneity for overall balance outcomes.

Outcomes	*K*	*N*	EG	CG	Hedges’ *g* [95%CI]	*I* ^*2*^	*Q*	*P*
TUGT	19	889	443	446	−0.89 [−1.20, −0.57]	79.26%	78.19	***
BBS	12	615	305	310	0.94 [0.70, 1.18]	47.80%	21.14	*
COP-EO	7	263	133	130	−0.66 [−0.97, −0.35]	32.72%	8.19	0.23
COP-EC	4	161	79	82	−0.34 [−0.65, −0.02]	1.19%	3.54	0.32

Note: TUGT, Timed Up and Go Test; BBS, Berg Balance Scale; COP-EO, Center of Pressure with Eyes Open; COP-EC, Center of Pressure with Eyes Closed; *K*, number of independent comparisons; *N*, total sample size; EG, experimental group; CG, control group; CI, confidence interval; *I*^*2*^, Higgins’ statistic for heterogeneity; *Q*, Cochran’s *Q* statistic; *P*, probability value for the *Q* test. *** P < 0.001, * P < 0.05.

For static balance control, data from 12 studies focusing on BBS scores (N = 615) were extracted for synthesis [8,47–49,51,53,56,58,59,62,65,66]. Compared to dynamic balance metrics, the analysis confirms that the interventions effectively enhance static stability in older adults (Hedges’ g = 0.94, 95% CI: 0.70, 1.18). The corresponding orchard and caterpillar plots display a more convergent data cluster distribution (Figs A4–A6 in S1 Appendix), with heterogeneity testing yielding moderate levels (I^2^ = 47.80%, Q = 21.14, p = 0.03).

At the micro-adaptive level of neuromuscular control, analysis of the COP parameters further quantified the efficacy of central feedback regulation [45,56,57,59,60,65,67]. Under eyes-open (EO) conditions, the pooled results of seven studies (N = 263) demonstrate a significant reduction in postural sway amplitude within the intervention group (Hedges’ g = −0.66, 95% CI: −0.97, −0.35). Visual mapping via orchard and caterpillar plots (Figs A7–A9 in S1 Appendix) illustrates that the effect size clusters of the independent studies exhibit a relatively compact distribution around the pooled estimate, corroborating the low heterogeneity associated with this synthesis (I^2^ = 32.72%, Q = 8.19, p = 0.23). Under eyes-closed (EC) conditions, the four included studies (N = 161) consistently indicate a positive effect of the intervention on improving postural control (Hedges’ g = −0.34, 95% CI: −0.65, −0.02) [45,56,57,65]. The corresponding orchard and caterpillar plots visually represent the extreme convergence of this dataset toward the central main effect line (Figs A10–A12 in S1 Appendix). Furthermore, the data distribution for this metric reveals exceptionally high statistical homogeneity (I^2^ = 1.19%, Q = 3.54, p = 0.32), further validating the robustness of this conclusion.

### Subgroup analyses

#### Subgroup analysis of dynamic balance (TUGT).

To trace the potential sources of effect size variation in dynamic balance (TUGT), this study conducted subgroup analyses based on participants’ health condition, intervention total sessions, intervention total dose, and age categories. Additionally, orchard and caterpillar plots were utilized to visualize the distribution characteristics of local data clusters (Table A3 and Figs A13–A17 in S1 Appendix).

**Fig 3:**
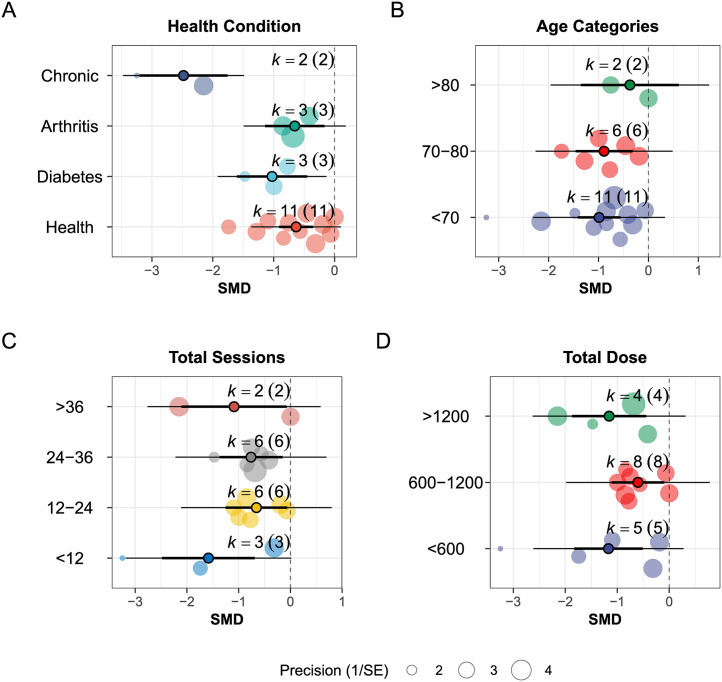
Orchard plots for subgroup analyses of TUGT performance categorized by (A) health condition, (B) age categories, (C) total sessions, and (D) total dose. TUGT, Timed Up and Go Test; *K*, number of independent comparisons; SMD, Standardized Mean Difference. Error bars represent the 95% confidence intervals (CI) and prediction intervals (PI).

Subgroup analysis confirmed that health status was a significant moderating variable driving heterogeneity (Q = 22.85, p < 0.001). Specifically, older adults with chronic diseases (Hedges’ g = −2.58, 95% CI: −3.63, −1.53, I^2^ = 66.3%) and diabetes (Hedges’ g = −0.99, 95% CI: −1.41, −0.58, I^2^ = 0.0%) demonstrated the greatest absolute magnitude of improvement. Conversely, healthy older adults (Hedges’ g = −0.64, 95% CI: −0.95, −0.32, I^2^ = 63.4%) and patients with joint pathologies (arthritis) (Hedges’ g = −0.66, 95% CI: −0.94, −0.38, I^2^ = 0.0%) exhibited a moderate yet stable enhancement in intervention benefits. The corresponding orchard and caterpillar plots visually illustrate (Fig 3A) that the healthy group data cluster is extensive with low dispersion, whereas the chronic disease group shows a substantial effect but with wider confidence intervals.

During the evaluation of intervention total sessions and total dose dimensions, one study was excluded from this subgroup analysis due to the lack of specific reporting on the intervention duration and frequency [55]. Following the exclusion of this dataset, no significant statistical differences were observed among the subgroups (Q = 3.20, p = 0.36). Short-term interventions comprising fewer than 12 sessions induced a higher initial effect (Hedges’ g = −1.71, 95% CI: −3.35, −0.07, I^2^ = 92.6%). When the frequency fell within the ranges of 12−24 sessions (Hedges’ g = −0.65, 95% CI: −0.99, −0.31, I^2^ = 47.4%) and 24−36 sessions (Hedges’ g = −0.70, 95% CI: −0.94, −0.45, I^2^ = 0.0%), the effect sizes retracted. The corresponding visual charts demonstrate highly overlapping confidence bands within these frequency intervals, indicating a convergence of heterogeneity (Fig 3C). Once interventions exceeded 36 sessions, the effect size exhibited an enhancing trend, although its confidence interval crossed the line of no effect (Hedges’ g = −1.08, 95% CI: −3.19, 1.03, I^2^ = 96.5%).

The stratified results for intervention total dose presented an evolutionary pattern highly isomorphic to the frequency dimension (Q = 2.45, p = 0.29); however, the internal trajectory of the data suggests potential non-linear adaptive characteristics. Specifically, both the low-dose (<600 minutes; Hedges’ g = −1.25, 95% CI: −2.29, −0.22, I^2^ = 91.2%) and high-dose (>1200 minutes; Hedges’ g = −1.16, 95% CI: −1.96, −0.36, I^2^ = 87.2%) intervals demonstrated significant intervention effects. In contrast, the effect size within the moderate-dose interval of 600−1200 minutes was relatively convergent (Hedges’ g = −0.60, 95% CI: −0.87, −0.32, I^2^ = 33.2%), with its orchard plot scatter distribution appearing most compact and closely aligned with the regression line (Fig 3D).

Evaluation of the age dimension revealed no significant statistical differences between groups (Q = 1.28, p = 0.53), yet the effect sizes exhibited a decreasing trend with advancing age (Fig 3B). Both the under-70 cohort (Hedges’ g = −1.00, 95% CI: −1.49, −0.51, I^2^ = 85.3%) and the 70–80 years cohort (Hedges’ g = −0.88, 95% CI: −1.32, −0.44, I^2^ = 66.5%) achieved significant improvements in dynamic balance. Conversely, the effect distribution for the over-80 subgroup shifted toward the null line, with the confidence interval crossing the line of no effect (Hedges’ g = −0.37, 95% CI: −1.11, 0.37, I^2^ = 66.5%). However, this over-80 subgroup was supported by only two studies; therefore, the apparent attenuation in this age stratum should be interpreted as an exploratory signal rather than a stable age-specific estimate. Overall, although health status significantly moderated the intervention effect on TUGT, substantial heterogeneity remained within several subgroups, particularly in the low-session, high-session, low-dose, and high-dose categories. This residual heterogeneity suggests that factors beyond the predefined participant- and dose-related variables may have contributed to the variability in TUGT effects. Potential intervention-level differences, such as surface stability, task complexity, progression strategy, dual-task components, and comparator intensity, were not consistently reported across the included studies and therefore could not be further examined in the present subgroup analysis.

#### Subgroup analysis of static balance (BBS).

For static postural control capacity (BBS), parallel subgroup analyses were conducted across identical demographic and clinical intervention dimensions (Table A4 and Figs A18–A22 in S1 Appendix). In contrast to the parameter sensitivity exhibited by the dynamic balance (TUGT) metric, the BBS demonstrated exceptional cross-group robustness across all stratifying variables.

**Fig 4:**
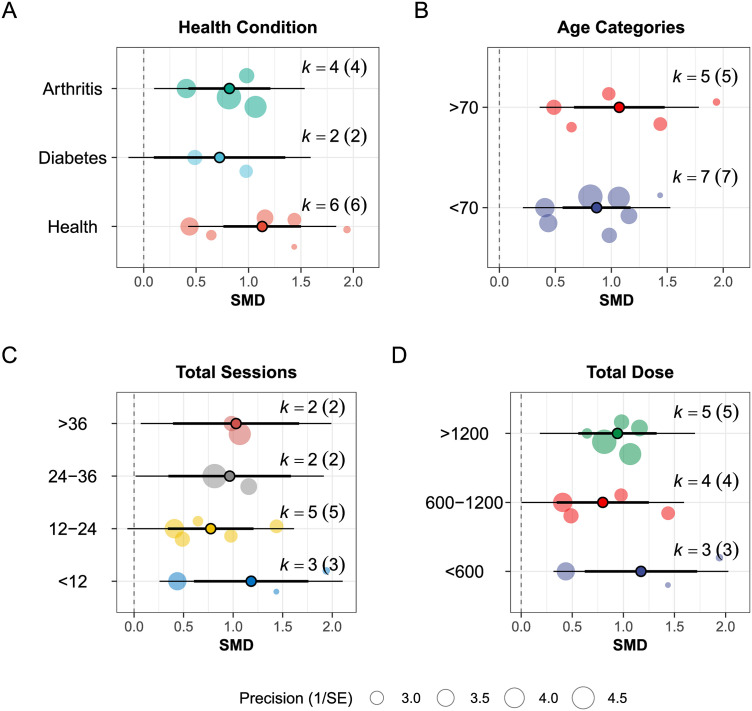
Orchard plots for subgroup analyses of BBS performance categorized by (A) health condition, (B) age categories, (C) total sessions, and (D) total dose. BBS, Berg Balance Scale; *K*, number of independent comparisons; SMD, Standardized Mean Difference. Error bars represent the 95% confidence intervals (CI) and prediction intervals (PI).

Within the health status dimension, tests for subgroup interactions revealed no significant statistical differences (Q = 1.86, p = 0.39). Quantitative data indicated that the healthy older adult cohort achieved significant gains in static balance (Hedges’ g = 1.14, 95% CI: 0.70, 1.59, I^2^ = 62.2%). Concurrently, participants in the arthritis group (Hedges’ g = 0.82, 95% CI: 0.55, 1.09, I^2^ = 19.1%) and the diabetes group (Hedges’ g = 0.72, 95% CI: 0.24, 1.20, I^2^ = 8.1%) also exhibited distinct positive intervention effects. Integrating the distribution characteristics from the visual charts (Fig 4A) reveals that the effect point estimates for all subgroups are robustly distributed to the right of the line of no effect, with the diabetes and arthritis subgroups demonstrating exceptionally high internal consistency.

Stratified assessments of intervention total sessions and total dose further substantiate the robustness of this outcome measure. Between-group differences were non-significant for both intervention total sessions (Q = 1.32, p = 0.72) and intervention total dose (Q = 1.06, p = 0.59). Specifically, because the subsets of studies comprising the short-term (< 12 sessions) and low-dose (< 600 minutes) interventions completely overlapped, both demonstrated a consistent and robust initial adaptive effect (Hedges’ g = 1.24, 95% CI: 0.34, 2.13, I^2^ = 79.8%). Upon entering the moderate intervention load interval, effect sizes exhibited a phase-specific retraction (12–24 sessions: Hedges’ g = 0.77, 95% CI: 0.39, 1.15, I^2^ = 43.1%; 600–1200 minutes: Hedges’ g = 0.80, 95% CI: 0.33, 1.27, I^2^ = 56.5%), and the scatter distribution in the corresponding charts displayed a more convergent trend (Fig 4C–D). Subsequently, within the 24–36 session frequency interval, the effect size began to steadily recover (Hedges’ g = 0.92, 95% CI: 0.59, 1.26, I^2^ = 0.0%). As intervention total sessions and dose continued to accumulate, static balance gains escalated again, exhibiting excellent homogeneity (>36 sessions: Hedges’ g = 1.04, 95% CI: 0.67, 1.41, I^2^ = 0.0%; > 1200 minutes: Hedges’ g = 0.94, 95% CI: 0.71, 1.18, I^2^ = 0.0%).

Analysis of the age dimension revealed no significant between-group differences (Q = 0.61, p = 0.43). Both the under-70 cohort (Hedges’ g = 0.86, 95% CI: 0.61, 1.11, I^2^ = 32.3%) and the older adult cohort aged over 70 (Hedges’ g = 1.08, 95% CI: 0.57, 1.59, I^2^ = 63.0%) achieved highly statistically significant improvements in static balance from the interventions. The corresponding orchard and caterpillar plots confirm a highly consistent response pattern to sensorimotor stimuli across different age strata (Fig 4B).

### Meta-regression analysis

#### Dose regression analysis.

**Fig 5:**
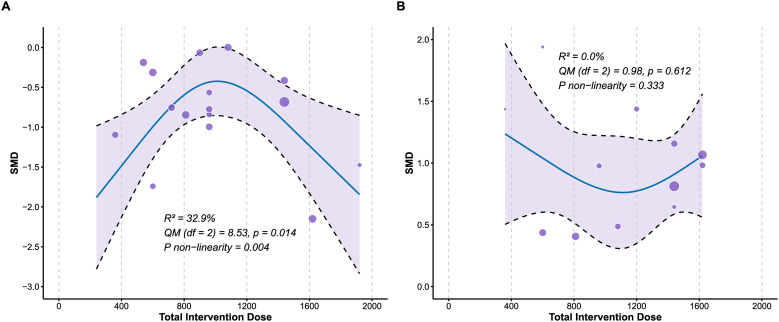
Non-linear dose-response meta-regression between total intervention dose and balance performance: (A) TUGT and (B) BBS. TUGT, Timed Up and Go Test; BBS, Berg Balance Scale; SMD, Standardized Mean Difference. The solid line represents the estimated dose-response curve, the shaded area represents the 95% confidence intervals (CI), and the dashed lines represent the upper and lower limits of the 95% CI.

This study employed a RCS model to deconstruct the dynamic moderating mechanisms of total intervention dose on balance performance improvements. Regarding dynamic balance (TUGT), one study was excluded from the dose regression analysis due to the lack of clearly reported quantitative parameters, such as intervention duration and frequency, which precluded the accurate calculation of its total intervention dose [55]. Following the exclusion of this missing data, the RCS regression model revealed a significant non-linear dose-response relationship between total intervention dose and effect size (P _non-linearity_ = 0.004) (Fig 5A). Model fitting statistics indicated (Q = 8.53, p = 0.014), R^2^ = 32.9%, demonstrating that the total dose variable can account for approximately 32.90% of the inter-study effect size variation (Table A5 in S1 Appendix). The dose-response fitted curve exhibited a distinct non-linear fluctuating trajectory: within the low-dose exposure interval (approximately 200–500 minutes) and the high-dose accumulation interval (approximately 1600–2000 minutes), the efficacy of sensorimotor-based interventions in improving TUGT peaked. Conversely, when the total dose fell within the moderate-load interval (approximately 1000 minutes), the effect size exhibited diminishing marginal returns.

In contrast, static balance (BBS) did not demonstrate significant dose-dependent characteristics. The RCS model fitting indicated that neither linear prediction nor non-linear association tests reached statistical significance (Q = 0.98, p = 0.612; P _non-linearity_ = 0.333) (Fig 5B). The dose regression trajectory for BBS remained flat, and the model’s explanatory power for heterogeneity was extremely low (R^2^ = 0.00%), suggesting that within the dose gradients covered by the current research, training duration is not a core covariate driving the divergence of static balance benefits (Table A6 in S1 Appendix).

#### Age regression analysis.

**Fig 6:**
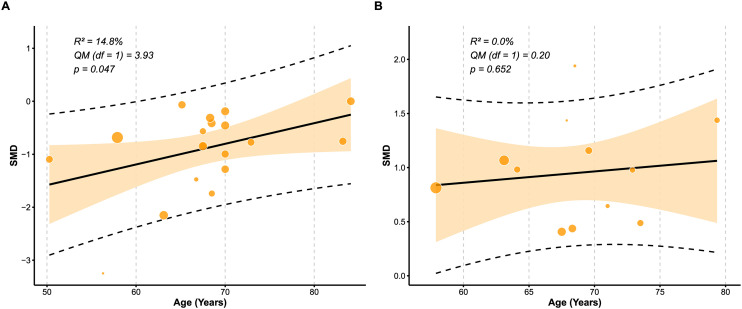
Linear meta-regression of age on balance performance: (A) TUGT and (B) BBS. TUGT, Timed Up and Go Test; BBS, Berg Balance Scale; SMD, Standardized Mean Difference. The solid line represents the meta-regression line, the shaded area represents the 95% confidence intervals (CI), and the dashed lines represent the upper and lower limits of the 95% prediction intervals (PI).

Building upon the investigation of intervention total dose effects on outcome measures, this study introduced a univariate random-effects meta-regression model to deconstruct the moderating role of participants’ baseline age on the magnitude of balance benefits. Regarding dynamic balance (TUGT), the meta-regression model confirmed that age is a crucial demographic covariate driving inter-study heterogeneity (Q = 3.93, p = 0.047, R^2^ = 14.77%), accounting for approximately 14.77% of the variance in effect size. Fitting results indicated a predicted regression coefficient of 0.039 (95% CI: 0.001, 0.078), suggesting that for each one-year increase in baseline age, the effect size of sensorimotor-based interventions on TUGT improvement linearly attenuates by 0.039 units (Table A7 in S1 Appendix). The bubble plot clearly maps the trajectory of the effect size shifting toward the null line as the age gradient increases (Fig 6A). This evidence suggests a potential constraint on neuromuscular remodeling potential in populations of advanced age, although the marginal significance warrants a cautious interpretation.

In contrast, static balance (BBS) exhibited high stability against age variations. Meta-regression analysis revealed no statistically significant linear association between baseline age and BBS effect size (Q = 0.20, p = 0.652, R^2^ = 0.00%). The predicted regression coefficient was merely 0.011 (95% CI: −0.035, 0.056). Data points in the bubble plot were discretely scattered on both sides of the regression line (Fig 6B), confirming the homogeneity of the intervention effect across different age strata (Table A8 in S1 Appendix).

Synthesizing the regression analysis results of the dose-response relationship and demographic characteristics, the remodeling effect of sensorimotor-based interventions on balance performance in older adults presents a differential moderation pattern: dynamic balance exhibits significant non-linear dose dependency and age sensitivity, whereas static balance demonstrates greater evidence-based robustness against the perturbations of multidimensional covariates.

### Publication bias and sensitivity analysis

**Fig 7:**
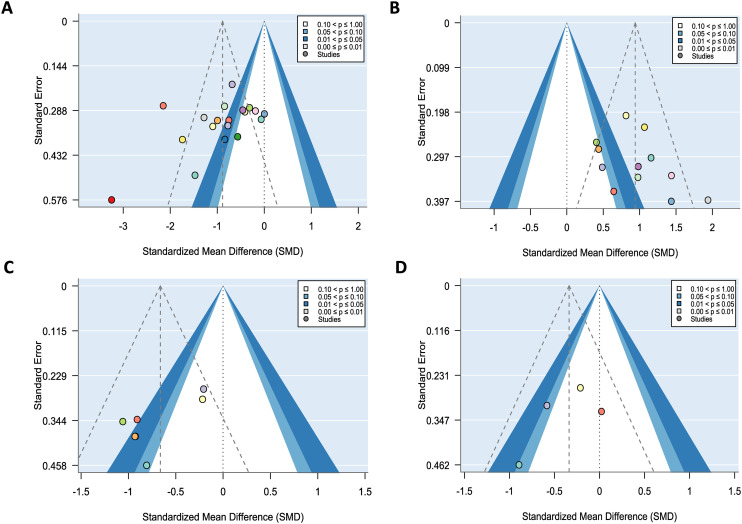
Contour-enhanced funnel plots for publication bias assessment: (A) TUGT, (B) BBS, (C) COP-EO, and (D) COP-EC. TUGT, Timed Up and Go Test; BBS, Berg Balance Scale; COP, Center of Pressure; COP-EO, Center of Pressure with Eyes Open; COP-EC, Center of Pressure with Eyes Closed. The shaded regions represent different levels of statistical significance.

To verify the reliability of the evidence synthesis and evaluate the impact of potential small-study effects on the pooled estimates, this study conducted a dual examination of publication bias utilizing contour-enhanced funnel plots (Fig 7A-D) and Egger’s linear regression test (Table A9 in S1 Appendix). Regarding dynamic balance (TUGT), Egger’s test detected a significant bias signal (p = 0.005), and the contour-enhanced funnel plots exhibited a clear asymmetric distribution pattern. To mitigate the potential perturbation of conclusions by this bias, the non-parametric trim-and-fill method was further employed to perform virtual iterations on the funnel plots (Figs A23–A24 in S1 Appendix). Following the identification and imputation of potentially missing studies, the adjusted pooled effect size for TUGT was Hedges’ g = −0.61 (95% CI: −1.01, −0.22). Although the adjusted confidence interval remained to the left of the line of no effect, indicating that the positive direction of the intervention benefit is maintained, the noticeable reduction in the effect size (from Hedges’ g = −0.89 to −0.61) suggests that the initial pooled estimate was likely overestimated due to small-study effects or publication bias. Therefore, the magnitude of improvement in dynamic balance should be interpreted with prudence. Concurrently, the neural adaptation metric under COP-EO also demonstrated a statistical tendency toward bias (Egger’s test, p = 0.024). Following trim-and-fill adjustment, the pooled effect size shifted from Hedges’ g = −0.66 to −0.78 (95% CI: −1.10, −0.46). While the direction and significance of the effect remained consistent pre- and post-correction, the presence of bias necessitates a more cautious interpretation of the specific effect magnitude. In comparison, the assessment results for static balance (BBS) and COP-EC demonstrated excellent stability. Egger’s test for the BBS revealed no significant statistical bias (p = 0.105), and data points within the contour-enhanced funnel plots were symmetrically and evenly distributed across the significance contour regions. COP-EC exhibited similar homogeneous characteristics (Egger’s test, p = 0.259), indicating an extremely low risk that this metric is influenced by publication bias.

Furthermore, the leave-one-out sensitivity analysis confirmed that the removal of any single study did not exert a directional impact on the pooled estimates for the four outcome measures (Figs A25–A28 in S1 Appendix). This finding indicates that the overall direction of the meta-analysis conclusions is not heavily driven by individual extreme data points, despite the necessary conservative interpretation regarding the exact magnitude of the benefits.

### Summary of evidence certainty

Appraisal of the evidence using the GRADE framework revealed a distinct gradient in certainty across the outcomes of sensorimotor-based interventions for balance performance in older adults (Table A10 in S1 Appendix). Static balance, assessed via the BBS, yielded a high-certainty rating (Hedges’ g = 0.94). This high level of confidence indicates that the estimated effect size is robust and serves as a reliable proxy for the true biological impact of the intervention, offering a stable foundation for clinical decision-making. In contrast to the high confidence observed in static stability, neuromuscular control under COP-EC was adjudicated as moderate certainty. This classification was necessitated by a single-tier downgrade for imprecision, as the aggregate sample size (N = 161) fell short of the optimal information size required to ensure high stability of the effect estimate (Hedges’ g = −0.34). Consequently, while the intervention appears beneficial, future research may influence the confidence in this specific estimate. At the lowest end of this evidence gradient, the certainty for dynamic balance (TUGT) and neuromuscular control under COP-EO was further attenuated to a low level. For dynamic balance (Hedges’ g = −0.89), the reduction was driven by the convergence of serious inconsistency (I^2^ = 79.26%) and significant publication bias detected through Egger’s test (p = 0.005). Similarly, the low certainty for COP-EO (Hedges’ g = −0.66) was attributable to the interplay between serious imprecision (N = 263) and suspected publication bias (p = 0.024). These findings suggest that for these specific parameters, current effect estimates may diverge substantially from the true underlying effect, underscoring the urgent need for large-scale, high-quality randomized controlled trials to bolster the existing evidence base.

## Discussion

### Summary of main findings

Synthesizing evidence across varying levels of certainty, this systematic review and meta-regression analysis robustly corroborates the therapeutic efficacy of sensorimotor-based paradigms—encompassing ST, PT, and PNF—in remodeling postural control within aging populations. Transcending conventional efficacy assessments, the strategic application of a RCS model provides a novel quantitative framework, explicitly delineating the fundamental divergence between the adaptive trajectories of dynamic and static balance.

An in-depth examination of the data reveals the complex non-linear dynamics of the body’s response to intervention loads. For dynamic balance, assessed via the TUGT, the model suggests a fluctuating adaptive continuum. An initial functional surge during early exposure (200–500 minutes) is likely driven by synaptic facilitation; as the cumulative load approaches the 1000-minute threshold, a phasic retraction of effect sizes emerges, indicating a saturation of continuous linear gains. Subsequent load accumulation (1600–2000 minutes) initiates deeper structural neuromuscular remodeling. However, these dynamic temporal fluctuations must be interpreted judiciously, as the underlying evidence is constrained to a low certainty level, primarily attenuated by substantial inter-study inconsistency and suspected publication bias. Conversely, static stability (BBS) exhibits robust homeostatic resilience across varying doses. Crucially, this flat regression trajectory is substantiated by high-certainty evidence, precluding simple temporal accumulation as the primary driver of efficacy. This integration of rigorous evidence quality confirms the exceptional, highly reliable stability of sensorimotor stimuli in preserving the base of support.

Deconstructing the modulatory influence of baseline characteristics exposes a divergent regulatory role for chronological age. Advanced age operates as a potential rate-limiting covariate for dynamic functional adaptation, reflected by a marginal linear attenuation in performance gains among older cohorts. Within the static dimension, however, aging nervous systems retain substantial neuroplastic reserves, exhibiting a resilient homeostatic capacity that withstands age-related degradation. While these macro-level static benefits are highly certain, the underlying micro-adaptive neuromuscular mechanisms—reflected by center of pressure parameters—are supported by moderate (eyes-closed) to low (eyes-open) certainty evidence, suggesting that specific sensory reweighting processes require further large-scale validation. Concurrently, distinct baseline pathological profiles profoundly dictate the predictability of rehabilitation trajectories. Cohorts presenting with metabolic impairments or structural alterations, notably diabetes or arthritis, demonstrate an exceptionally homogeneous response signature lacking statistical heterogeneity (I^2^ = 0.0%). This uniformity implies that compromised postural control systems maintain a highly consistent and verifiable receptivity to sensorimotor stimuli, reinforcing the clinical viability of this intervention paradigm.

Underpinning the reliability of these clinical observations, rigorous sensitivity simulations and non-parametric trim-and-fill adjustments systematically mitigated the influence of suspected publication biases on the estimated effect sizes. By integrating these methodologically safeguarded non-linear dose-response trajectories with the established evidence certainty ratings, the current study constructs a hierarchical theoretical framework. This paradigm explicitly dismantles the homogenized “more is better” rehabilitation fallacy, thereby establishing certainty-stratified, quantitative benchmarks for designing precise exercise prescriptions tailored to specific physiological thresholds, underlying pathologies, and age-related adaptive capacities.

### Comparison with previous studies

While the overarching conclusions regarding the efficacy of sensorimotor-based interventions align with recent high-quality systematic reviews—reaffirming the positive remodeling impact of neuromuscular adaptation on postural control in older adults—substantial methodological distinctions elevate the current study. These advancements manifest primarily in the dimensional approach to scientific inquiry and the analytical depth of the established evidence chain.

Previous systematic reviews and meta-analyses have shown that sensorimotor, proprioceptive, balance-oriented, and multicomponent exercise interventions can improve balance performance, functional mobility, and fall-related outcomes in older adults [17]. However, these studies have mainly addressed overall efficacy, intervention type, or broad prescription variables, rather than the non-linear cumulative dose-response pattern of sensorimotor-based interventions [18]. Therefore, their clinical implications support the general use of balance-related exercise, but provide limited guidance on how training dose should be adjusted when functional improvement begins to slow. The present study extends this evidence by showing that dynamic balance and static balance may follow different dose-response trajectories, suggesting that clinical prescription should consider the target outcome, cumulative dose, and the need for task progression rather than simply increasing total training duration.

The transition from “horizontal modality selection” to “longitudinal dose optimization” constitutes a critical breakthrough for this study within the evidence-based framework. Current mainstream paradigms, primarily represented by network meta-analyses, anchor on the cross-sectional efficacy comparison among different intervention modalities, aiming to construct a hierarchical pyramid of intervention strategies [2,68]. Although this modality-centric perspective resolves qualitative clinical queries regarding therapeutic superiority, it inherently neglects the longitudinal dimension of time accumulation. Consequently, traditional models fail to elucidate how training load, functioning as a continuous variable, dictates non-linear qualitative transformations in physiological adaptation. Bridging this quantitative gap, the current application of an RCS model maps the non-linear saturation trajectory of dynamic balance, explicitly capturing a critical phase of diminishing returns near the 1000-minute exposure threshold. Transcending the constraints of linear dose-accumulation hypotheses, this inflection point offers a pragmatic efficiency metric for clinical practice. Upon traversing this temporal boundary, relying exclusively on prolonged training durations may yield diminishing marginal functional gains, suggesting that integrating dynamic progressions in task complexity represents a superior strategy for optimizing prescription cost-effectiveness.

Additionally, this study achieved an evolutionary leap in the evidence chain, transitioning from qualitative confirmation to macro-micro mutual verification. Although previous traditional meta-analyses extensively examined the intervention sensitivity of various outcome measures [7], they remained confined to confirming the significance of overall efficacy within the dose dimension. This approach masks the complex non-linear dynamics inherent in the physiological adaptation process and fails to ascertain why certain high-dose interventions do not trigger expected additional benefits. Overcoming this limitation, the current study moves beyond the singular reliance of previous literature on macroscopic clinical phenotypes (e.g., TUGT, BBS) by systematically incorporating microscopic trajectory parameters of the COP [69]. Synthesizing these biomechanical metrics through the GRADE framework provides a transparent and nuanced understanding of the underlying neural mechanisms. Specifically, optimizations in postural control under COP-EC are supported by moderate-certainty evidence and near-perfect statistical homogeneity (I^2^ = 1.19%), offering relatively reliable substantiation for the sensory reweighting mechanisms induced by sensorimotor stimuli. In contrast, while micro-adaptive improvements are observed under COP-EO, these specific findings must be interpreted with academic prudence, as their evidence base is constrained to a low certainty level by sample size imprecision and suspected publication biases. Despite these varying gradations of certainty, this multidimensional corroborative network successfully bridges macroscopic functional performance with microscopic biomechanical mechanisms. This dual perspective establishes a profound neurophysiological baseline, far exceeding conventional clinical assessments, for deciphering the boundaries of plasticity reserves within the aging nervous system.

### Divergence in dose-response: Mechanisms of adaptation

This investigation maps a complex adaptive landscape of the aging neuromuscular system when navigating various postural challenges. Regarding dynamic balance (TUGT), the RCS regression maps a significant non-linear evolution (P non-linearity = 0.004), delineating a dynamic physiological trajectory progressing from synaptic facilitation to structural remodeling. During the early exposure window (approximately 200–500 minutes), substantial functional gains likely stem from synaptic facilitation typical of early motor learning; specifically, the central nervous system achieves performance leaps by optimizing the recruitment rate and synchronization of motor units [4,70]. However, as the cumulative load approaches the 1000-minute node, a relative attenuation in benefits reflects a potential phase of diminishing marginal returns rather than continuous adaptation. This pattern is consistent with the dose-stratified findings, in which the pooled effect was larger in the low-dose interval (<600 minutes; g = −1.25) and high-dose interval (>1200 minutes; g = −1.16), but attenuated in the moderate-dose interval of 600–1200 minutes (g = −0.60). Rather than indicating a fixed neural saturation point, the 1000-minute plateau may represent a transitional adaptive bottleneck, where repeated exposure to similar sensorimotor stimuli becomes less efficient after early gains have been achieved. For clinicians, this inflection should be interpreted as a signal to progress task complexity, not merely to extend training duration. Increasing sensory challenge, reducing visual dependence, introducing unstable surfaces, incorporating directional changes, or adding cognitive-motor dual-task demands may help renew the adaptive stimulus and support further improvements in dynamic balance. As interventions extend into the high-load interval (1600–2000 minutes), the subsequent escalation in efficacy implies a transition into deep adaptation, dominated by cortical structural remodeling and the consolidation of sensory reweighting networks [71,72]. Crucially, given the low-certainty evidence constraining the underlying dynamic balance metrics, these precise temporal thresholds represent a highly biologically plausible hypothesis rather than an immutable definitive pathway, warranting prudent clinical interpretation.

The substantial heterogeneity observed in TUGT further underscores the need for caution when interpreting this dynamic balance trajectory. Although health status emerged as a significant moderator in the subgroup analysis, this factor alone is unlikely to account for the full dispersion of effects. TUGT is a functionally integrated measure rather than a pure balance task, requiring sit-to-stand transition, gait initiation, forward walking, turning, deceleration, and return-to-sitting control within a single timed sequence [73]. As a result, its responsiveness may be especially sensitive to subtle differences in intervention design. Across the included trials, sensorimotor-based programs varied not only in overall dose but also in the nature of the training stimulus. Exercises performed on unstable surfaces, foam pads, balance boards, Swiss balls, or other proprioceptively enriched environments may impose greater sensory reweighting and postural-control demands than training conducted primarily on stable ground [74]. Likewise, programs incorporating dual-task elements, directional changes, reactive stepping, or progressive task complexity may transfer more directly to TUGT performance than single-task or predominantly static protocols [75]. Comparator conditions also differed across studies, ranging from no training to conventional therapy or active exercise controls, which may have further magnified between-study variation. These intervention-level differences were not reported consistently enough to permit reliable subgroup modeling, but they likely contributed to the residual heterogeneity surrounding the pooled TUGT effect. Therefore, the observed benefit for dynamic balance should be interpreted as a general direction of effect rather than a uniform response across all sensorimotor-based prescriptions. This interpretation is also supported by the publication-bias results. Egger’s test showed significant asymmetry for TUGT, and the trim-and-fill procedure reduced the pooled effect from g = −0.89 to g = −0.61. Although the adjusted estimate still favored sensorimotor-based interventions, this attenuation suggests that the current literature may overestimate their benefits for dynamic agility and functional mobility.

Conversely, the adaptive mechanisms underpinning static balance (BBS) are firmly anchored by high-certainty evidence. Although the RCS model precludes significant continuous dose-dependent characteristics (P non-linearity = 0.333), the fluctuations in effect sizes across discrete dose intervals provide an essential supplementary perspective for exploring these highly reliable adaptive shifts. In the low-dose phase (<600 minutes), the rapid establishment of static stability likely depends on the swift arousal of multisensory integration mechanisms, wherein the nervous system acutely corrects postural deviations by recalibrating sensory input weights [5,76]. Entering the moderate-dose interval (600–1200 minutes), the convergence in improvement magnitude suggests that feedback loops encounter physiological habituation, characterized by adaptive desensitization of the central nervous system to highly predictable stimuli [77]. The robust ascent in effect size during the high-dose phase (>1200 minutes) indicates a profound qualitative shift in postural control strategy, transitioning from cortical cognitive compensation to subcortical network automation [13,72]. At this stage, accumulating load likely assists the organism in overcoming mid-term adaptive bottlenecks, driving the central nervous system to transfer stabilization tasks to low-cognitive-demand automated networks, thereby achieving the structural consolidation of static balance gains [71].

This isomorphism between the divergent statistical manifestations of dose regression and the underlying biological trends profoundly illustrates that the aging central nervous system likely adheres to a convergent basal neural adaptation logic when processing postural control tasks of varying complexity. Nevertheless, this overarching theoretical synthesis warrants cautious interpretation, given the distinct gradations of evidence certainty characterizing each specific functional domain.

### Moderating factors: Age & neural plasticity

Having elucidated the phase-specific mechanistic evolution driven by intervention dose, further exploring the moderating role of individual heterogeneity on this neural remodeling process becomes particularly crucial. As a fundamental covariate constraining neuromuscular adaptation, biological age exhibits divergent moderating trajectories across different dimensions of balance remodeling. Current findings indicate that advancing age marginally attenuates dynamic balance benefits, assessed via the TUGT (β = 0.039, P = 0.047). This trend likely reflects the heavy reliance of dynamic postural control on rapidly depleting neuromuscular reserves. The aging-induced decline in type II fast-twitch muscle fiber recruitment and the slowing of spinal motor neuron conduction inherently limit the capacity for rapid motor planning in populations of advanced age. Because the overarching evidence for dynamic balance is constrained to a low certainty level, and data for specific cohorts over 80 remain sparse, inferences regarding these physiological restrictions on dynamic functional transformations demand highly cautious, evidence-grounded interpretation [78,79].

In stark contrast to the age-related constraints observed in dynamic performance, the “cross-age universality” demonstrated by static balance (BBS) provides a highly resilient window for geriatric rehabilitation. Supported by high-certainty evidence, the maintenance of static homeostasis emphasizes the central integration of minute deviation signals rather than explosive power output; thus, the cerebellar and basal ganglia circuits responsible for sensory processing appear to retain substantial plasticity potential, even in cohorts aged over 70. This divergence underscores a robust homeostatic resilience within the aging nervous system, suggesting that its remodeling mechanisms stem more from enhanced sensory integration efficiency than from improved peripheral muscle strength.

This adaptive paradigm, fundamentally predicated on sensory reweighting, provides critical evidence for understanding the plasticity boundaries of the aging nervous system. Micro-level biomechanical analyses further illuminate this mechanism, albeit with varying degrees of evidentiary confidence. In-depth analysis of COP metrics reveals that the significant advantage of sensorimotor-based interventions in optimizing postural sway under COP-EO confirms that the complex interaction among visual, vestibular, and proprioceptive inputs is a prerequisite for inducing neural reorganization in the aging brain [5,80,81]; however, this specific finding is currently limited by low-certainty evidence. Conversely, the attenuated magnitude of improvement exhibited under COP-EC, grounded in moderate-certainty evidence, exposes the functional compensatory limitations of the aging central nervous system following the withdrawal of visual compensation: forcing an attenuated nervous system to rely exclusively on proprioception for homeostatic remodeling essentially constitutes a high-load neurocognitive challenge [76,77]. This finding deepens the fundamental understanding of age effects, indicating that the adaptation of older populations to complex postural tasks is constrained not only by the deterioration of peripheral executive apparatuses but also by the compensatory elevation of central integration thresholds. Recognizing these distinct physiological compensatory boundaries provides a highly nuanced, micro-control rationale for designing precise, capability-matched rehabilitation interventions.

### Clinical implications

Translating these non-linear dose-response dynamics into precise clinical algorithms provides a core pivot for advancing evidence-based geriatric rehabilitation. Current findings indicate that dynamic balance remodeling, as assessed by TUGT, may encounter a phase of diminishing marginal returns when the cumulative intervention dose approaches approximately 1000 minutes. While this threshold emerges as a prominent statistical inflection point, its clinical application should be framed by the varying gradations of evidence certainty identified in this study. Accordingly, the 1000-minute node should not be interpreted as a rigid prescription boundary, but as a practical decision point at which clinicians should reassess whether continued exposure to the same sensorimotor tasks remains sufficient. Consequently, clinicians are encouraged to move beyond a homogenized “more is better” dosing mindset and adopt a bimodal, function-oriented prescription strategy to optimize rehabilitation benefits while reducing inefficient dose accumulation during the plateau phase.

In this framework, the term “bimodal” refers to two clinically meaningful dose windows identified from the non-linear trajectory: an early low-dose window that favors rapid functional gains and a later high-dose window that may support deeper postural consolidation. The term “polarized” indicates that prescription decisions should be oriented toward different clinical goals according to the older adult’s functional profile, rather than centered on a single average dose. For populations constrained by limited rehabilitation resources, reduced adherence, or the need for rapid improvement in functional mobility, prescription design should emphasize the left-shifted “agile benefit” model. This model focuses on the early response window (<600 cumulative minutes), aiming to capture meaningful gains in dynamic mobility and daily functional transitions with a relatively low time burden. Clinically, this approach may be most suitable for older adults with relatively preserved mobility, early balance decline, or mild functional slowing, where the primary goal is to improve sit-to-stand transition, gait initiation, turning, and multidirectional stepping.

Conversely, for older adults with persistent postural instability, high fall risk, complex environmental demands, or the need for durable stabilization, the prescription focus may shift toward the right-sided “deep consolidation” model. This approach intentionally extends beyond the 1000-minute inflection point and emphasizes progressive sensory and motor complexity rather than simple repetition of the same tasks. In this model, the clinical priority is to consolidate postural control through sensory reweighting, reduced visual dependence, unstable or compliant surfaces, reactive stepping, directional changes, perturbation-based tasks, and cognitive-motor dual-task demands. However, given that the evidence for dynamic balance and microscopic postural control, particularly TUGT and COP-EO, remains at a low certainty level, these long-term structural remodeling recommendations should be viewed as clinically plausible trajectories that require individualized monitoring rather than definitive prescriptions.

The intermediate dose zone, approximately 600–1200 cumulative minutes, deserves particular clinical attention. This interval should not be interpreted as ineffective; instead, it may represent a period in which repeated exposure to familiar sensorimotor stimuli produces fewer additional gains. In practice, slowed progress within this zone should prompt clinicians to reassess task difficulty, progression rules, and the match between training stimuli and functional goals. Rather than merely extending session number or total duration, clinicians may need to renew the adaptive stimulus by increasing sensory challenge, adding turning or stepping transitions, reducing visual reliance, or introducing dual-task conditions. Table 3 provides a practical framework for applying this bimodal prescription strategy according to the older adult’s clinical profile and rehabilitation goal.

**Table 3:** Practical framework for applying the bimodal prescription strategy in older adults.

Prescription model	Suggested cumulative dose	Suitable older adult profile	Primary clinical goal	Training focus	Practical implementation
Agile benefit model	<600 min	Older adults with early balance decline, mild functional slowing, relatively preserved mobility, limited rehabilitation time, or lower adherence capacity	Improve functional mobility and dynamic balance within a relatively short intervention period	Sit-to-stand transition, gait initiation, turning, multidirectional stepping, and moderate sensory challenge	Begin with stable or semi-stable conditions. Use short-to-moderate sessions focused on task-specific movements such as standing up, initiating gait, turning, and stepping. Add speed variation or simple directional changes when tolerated.
The intermediate dose zone	600–1200 min	Older adults whose improvement slows after early gains or who appear adapted to repeated tasks	Determine whether continued repetition is sufficient or whether task difficulty should be progressed	Task progression, sensory challenge, and functional specificity	Monitor TUGT-related components, including sit-to-stand, gait transition, and turning. Avoid only increasing session number. Progress the stimulus by adding unstable surfaces, reduced visual input, directional changes, or simple cognitive-motor dual tasks when appropriate.
Deep consolidation model	>1000 min or >1200 min	Older adults with persistent postural instability, recurrent balance deficits, higher fall risk, strong visual dependence, or difficulty managing complex daily environments	Support longer-term stabilization of postural control and sensory integration	Progressive proprioceptive challenge, unstable or compliant surfaces, reduced visual reliance, reactive stepping, perturbation-based tasks, and cognitive-motor dual-task demands	Progress from repeated sensorimotor exposure to more challenging tasks. Use foam pads, balance boards, unstable surfaces, reactive stepping, turning with reduced visual input, and dual-task walking or stepping. Adjust progression according to fatigue, safety, and functional response.
High-caution subgroup	Individualized	Adults over 80 years old, frail individuals, or those with substantial comorbidity, pain, fear of falling, or low exercise tolerance	Maintain safety while supporting gradual improvement	Conservative progression, close supervision, fall-risk control, and individualized task selection	Start below the individual’s instability threshold. Prioritize supervised training, stable support options, and gradual sensory progression. Avoid rapid increases in instability or dual-task load unless safety and tolerance are confirmed.

The clinical utility of this stratification model is further enhanced by the exceptional response homogeneity (I^2^ = 0.0%) observed in specific pathological cohorts, such as those with diabetes or arthritis. This indicates that for structurally or metabolically compromised systems, sensorimotor-based interventions offer a highly predictable therapeutic pathway. This precise stratification profoundly reflects the applied value of precision exercise medicine within geriatric care [3,21]. Simultaneously, clinical practice must astutely identify individual biological constraints. Given the limited evidence available for adults over 80 years old, clinicians should not interpret the observed attenuation as a definitive age-related ceiling. Instead, this subgroup should be approached with more conservative progression, closer monitoring, and individualized balancing between agile improvement and long-term stabilization. By avoiding blind investments within the saturation zone, this polarized decision-making paradigm significantly enhances the allocative efficiency of medical resources while ensuring that intervention protocols precisely target optimal clinical efficacy and safeguard biological safety.

### Limitations and future research

The current study, despite its rigorous methodological framework, is not without inherent constraints that warrant a balanced and cautious interpretation. The interpretative depth of the observed dose-response trajectories is fundamentally bounded by the varying gradations of evidence certainty identified through the GRADE framework. Specifically, while the benefits for static balance are substantiated by high-certainty data, the evidence for neuromuscular control under COP-EC reaches moderate certainty, whereas findings for dynamic balance (TUGT) and postural sway under COP-EO remain constrained to a low certainty level. This evidentiary stratification, driven primarily by substantial inter-study inconsistency and suspected publication biases, suggests that the identified 1000-minute saturation threshold should be generalized with considerable academic prudence. The observed dispersion in effect sizes may reflect unmeasured variances in the specific biomechanical loads of ST, PT, and PNF, which further necessitates a cautious contextualization of the quantitative findings. An additional limitation concerns the exclusion of non-English literature. Although the included trials represented a geographically diverse evidence base, including studies from Brazil, Iran, South Korea, Pakistan, Spain, Turkey, and other regions, eligible trials published only in local languages may have been missed. This restriction may have introduced linguistic bias, particularly if local-language studies differed from English-language publications in participant characteristics, intervention delivery, rehabilitation settings, or reported effect sizes. Therefore, the generalizability of the present findings to the global older-adult population should be interpreted with caution.

Furthermore, findings for the oldest-old subgroup should be interpreted with particular caution. Although participants over 80 years old showed attenuated dynamic balance benefits, this result was based on only two studies and should be considered preliminary. The shift toward the null line may indicate reduced adaptive capacity in very old adults, but it may also reflect small sample size, comorbidity burden, baseline functional differences, or variation in intervention protocols. Thus, the present findings should not be taken as definitive evidence of an age-related physiological ceiling. Future large-scale RCTs should recruit sufficient numbers of oldest-old participants to clarify whether age independently modifies the dose-response trajectory of sensorimotor-based interventions. Parallel to these evidence-level constraints, the reliance on macroscopic clinical phenotypes in the absence of direct neurophysiological monitoring, such as fMRI or fNIRS, renders the proposed mechanisms of subcortical automation and sensory reweighting largely deductive. Future dose-response trials should therefore incorporate brain plasticity biomarkers to determine whether the 1000-minute threshold corresponds to measurable cortical or subcortical reorganization. These biomarkers may include sensorimotor cortical activation, prefrontal recruitment, functional connectivity, cortical thickness, white matter integrity, or task-evoked hemodynamic responses assessed using fMRI, fNIRS, EEG, or related neuroimaging approaches. Such data would help clarify whether the observed clinical plateau reflects neural saturation, insufficient task progression, or a transition toward deeper structural adaptation. This epistemic gap is exacerbated by the restricted temporal scope of existing literature, which predominantly focuses on 4-to-18-week paradigms.

To bridge these gaps, future research should prioritize large-scale, adequately powered, pre-registered RCTs with standardized sensorimotor protocols, transparent dose reporting, and prespecified outcome hierarchies. This need is particularly urgent for outcomes currently supported by low-certainty evidence, including dynamic balance assessed by TUGT and neuromuscular control under eyes-open conditions assessed by COP-EO. Such trials are necessary to determine whether the observed non-linear TUGT trajectory and COP-EO response pattern represent reproducible dose-response phenomena, rather than unstable estimates shaped by small samples, inter-study inconsistency, or publication bias. Future systematic reviews should also consider multilingual searches and translation-assisted screening to capture evidence from a broader range of cultural, clinical, and rehabilitation contexts. Integrating high-resolution brain function monitoring with longitudinal tracking will be essential to transition from inferential mechanisms to a verified understanding of neural topological evolution across the aging lifespan. Moreover, future investigations should explore whether the dynamic progression of task complexity can effectively transcend the identified physiological bottlenecks, ensuring that the transition from quantitative load accumulation to qualitative neural adaptation is both sustained and optimized within clinical geriatric practice.

## Conclusions

This study provides evidence that sensorimotor-based interventions significantly improve balance performance in older populations, although the strength of these conclusions varies across balance dimensions according to the certainty of the evidence. Improvements in static balance (BBS) are supported by high-certainty evidence, demonstrating exceptional stability and no significant dose-dependency. In contrast, findings for dynamic balance (TUGT) and neuromuscular control (COP) should be interpreted with caution due to the lower certainty of the current evidence. These results suggest that dynamic balance exhibits significant non-linear adaptive characteristics, with neural benefits potentially experiencing a phase of diminishing marginal returns as the cumulative intervention dose approaches 1000 minutes. Furthermore, regression analysis indicates that biological age may exert a moderating effect; specifically, the magnitude of improvement in dynamic balance appears to attenuate with advancing age, whereas static stability remains resilient. Consequently, clinical practice must transcend homogenized paradigms to implement precise, stratified interventions that account for these non-linear dose-response patterns and the varying certainty of the evidence. Future large-scale, high-quality empirical studies are urgently required to further validate and refine this quantitative decision-making framework.

## Supporting information

S1 Checklist PRISMA 2020 Checklist. (PDF)

S1 Appendix Supplementary Material A. This file contains the complete search strategies, characteristics of included studies (A1-A2 Tables), overall meta-analysis results (A1-A12 Figs), subgroup analyses (A13-A22 Figs, A3-A4 Tables), meta-regression analysis (A5-A8 Tables), publication bias and sensitivity analyses (A9 Table, A23-A28 Figs), and certainty of evidence assessment using the GRADE framework (A10 Table).
(PDF)

S2 Appendix Supplementary Material B. Detailed results of the risk of bias assessment using the ROB2 tool.
(PDF)

S1 File Raw datasets and analytical codes. This compressed archive (ZIP) contains the original extraction forms, meta-analysis datasets (Excel format), and the R scripts utilized for all statistical analyses, including restricted cubic spline (RCS) and meta-regression.
(RAR)
