# Supplementary material for: Dose-response relationships of sensorimotor-based interventions on balance performance in older adults: A systematic review and meta-regression analysis
Source: PLoS One. 2026 Jul 23;21(7):e0354522. doi: 10.1371/journal.pone.0354522 (PMC13395370; doi:10.1371/journal.pone.0354522)
Supplement: S1 Appendix — This file contains the complete search strategies, characteristics of included studies (A1-A2 Tables), overall meta-analysis results (A1-A12 Figs), subgroup analyses (A13-A22 Figs, A3-A4 Tables), meta-regression analysis (A5-A8 Tables), publication bias and sensitivity analyses (A9 Table, A23-A28 Figs), and certainty of evidence assessment using the GRADE framework (A10 Table). (PDF) [file pone.0354522.s002.pdf]

Supplementary Material

Appendix A: Search Strategies 1

- Table A1. The complete search strategy for the databases

PubMed, Cochrane Library, Embase, Web of Science, and EBSCOhost

Appendix B: Study Characteristics 4

- Table A2. Characteristics of the included randomized controlled trials

Appendix C: Overall Meta-analysis Results 9

- Figs A1–A3. Overall effect on TUGT: Forest, Caterpillar, and Orchard plots
- Figs A4–A6. Overall effect on BBS: Forest, Caterpillar, and Orchard plots
- Figs A7–A9. Overall effect on COP-EO: Forest, Caterpillar, and Orchard plots
- Figs A10–A12. Overall effect on COP-EC: Forest, Caterpillar, and Orchard plots

Appendix D: Subgroup Analysis 16

- Fig A13. Combined caterpillar plots for TUGT subgroup analyses
- Figs A14–A17. Detailed subgroup forest plots for TUGT (Health, Sessions, Dose, Age)
- Fig A18. Combined caterpillar plots for BBS subgroup analyses
- Figs A19-A22. Detailed subgroup forest plots for BBS (Health, Sessions, Dose, Age)
- Table A3. Subgroup analysis results for Dynamic Balance (TUGT)
- Table A4. Subgroup analysis results for Static Balance (BBS)

Appendix E: Meta-regression Analysis 28

- Table A5. Non-linear Dose-response meta-regression results of TUGT
- Table A6. Non-linear Dose-response meta-regression results of BBS
- Table A7. Linear meta-regression results of baseline age on TUGT
- Table A8. Linear meta-regression results of baseline age on BBS

Appendix F: Publication Bias and Sensitivity Analysis 30

- Table A9. Summary of publication bias (Egger’s test) and sensitivity analysis
- Figs A23–A24. Trim-and-fill funnel plots for TUGT and COP-EO
- Figs A25–A28. Leave-one-out sensitivity analysis for TUGT, BBS, COP-EO, and COP-EC

Appendix G: Certainty of Evidence (GRADE Assessment) 33

- Table A10. GRADE level of evidence for this study’s findings

Appendix A: Search Strategies

Table A1. The complete search strategy for the databases

Database	Complete Search Strategy
PubMed	((("Aged"[Mesh] OR "Aged, 80 and over"[Mesh] OR "Frail Elderly"[Mesh] OR "Older Adults"[Title/Abstract] OR "Older People"[Title/Abstract] OR "Elderly"[Title/Abstract] OR "Senior*"[Title/Abstract] OR "Geriatric*"[Title/Abstract] OR "Old People"[Title/Abstract] OR "Community-Dwelling"[Title/Abstract]) AND (("Sensorimotor Exercise"[tiab] OR "Sensory Motor Training"[tiab] OR "Sensorimotor training"[tiab] OR "somatosensory exercises"[tiab] OR "Proprioceptive Exercise"[tiab] OR "Proprioceptive Training"[tiab] OR "Proprioceptive Neuromuscular Facilitation "[tiab] OR " Proprioceptive Neuromuscular Facilitation Technique "[tiab] OR "Proprioceptive Neuromuscular Facilitation Stabilization Techniques "[tiab] OR "PNF"[tiab])) AND ("Postural Balance"[Mesh] OR "Proprioception"[Mesh] OR "Electromyography"[Mesh] OR "balance control"[tiab] OR "postural stability"[tiab] OR "postural sway"[tiab] OR "center of pressure"[tiab] OR "COP"[tiab] OR "sway area"[tiab] OR "sway velocity"[tiab] OR "sensory organization test"[tiab] OR "SOT"[tiab] OR "muscle activation"[tiab] OR "neuromuscular control"[tiab] OR "EMG"[tiab] OR "Timed Up and Go"[tiab] OR "TUGT"[tiab] OR "Berg Balance Scale"[tiab] OR "BBS"[tiab])) AND ("Randomized Controlled Trial"[Publication Type] OR "Randomized Controlled Trials as Topic"[Mesh] OR "Randomized"[Title/Abstract] OR "Randomised"[Title/Abstract] OR "Placebo"[Title/Abstract] OR "Randomly"[Title/Abstract] OR "Trial"[Title/Abstract] OR "Groups"[Title/Abstract]) **107**
Cochrane Library	**#1** MeSH descriptor: [Aged] explode all trees **288785**<br>**#2** MeSH descriptor: [Aged, 80 and over] explode all trees **72594**<br>**#3** #1 OR #2 **288785**<br>**#4** (older adults):ti,ab,kw or (old people):ti,ab,kw or (senior):ti,ab,kw or (elders):ti,ab,kw or (geriatric):ti,ab,kw or (elderly):ti,ab,kw **106083**<br>**#5** #3 OR #4 **362158**<br>**#6** "Sensorimotor Exercise":ti,ab,kw OR "Sensory Motor Training":ti,ab,kw OR "Sensorimotor training":ti,ab,kw OR "somatosensory exercises":ti,ab,kw OR "Proprioceptive Exercise":ti,ab,kw OR "Proprioceptive Training":ti,ab,kw OR "Proprioceptive Neuromuscular Facilitation":ti,ab,kw OR "Proprioceptive Neuromuscular Facilitation Technique":ti,ab,kw OR "Proprioceptive Neuromuscular Facilitation Stabilization Techniques":ti,ab,kw OR "PNF":ti,ab,kw **2023**<br>**#7** MeSH descriptor: [Postural Balance] explode all trees **4747**<br>**#8** MeSH descriptor: [Proprioception] explode all trees **5565**<br>**#9** #7 OR #8 **5565**<br>**#10** (Postural Balance):ti,ab,kw or (Posture Balance):ti,ab,kw or (Balance, Posture):ti,ab,kw or (Postural Equilibrium):ti,ab,kw or (Equilibrium, Postural):ti,ab,kw or (Balance, Postural):ti,ab,kw or (Postural Control):ti,ab,kw or (Control, Postural):ti,ab,kw or (Postural Controls):ti,ab,kw or (Posture Control):ti,ab,kw or (Control, Posture):ti,ab,kw or (Posture Controls):ti,ab,kw or (postural sway):ti,ab,kw or (Posture Balances):ti,ab,kw or (Posture Equilibrium):ti,ab,kw or (Equilibrium, Posture):ti,ab,kw or (Posture Equilibriums):ti,ab,kw or

	(Musculoskeletal Equilibrium):ti,ab,kw or (Equilibrium, Musculoskeletal):ti,ab,kw or (postural stability):ti,ab,kw or (center of pressure):ti,ab,kw or (COP):ti,ab,kw or (sway area):ti,ab,kw or (sway velocity):ti,ab,kw or (sensory organization test):ti,ab,kw or (SOT):ti,ab,kw or (muscle activation):ti,ab,kw or (neuromuscular control):ti,ab,kw or (Timed Up and Go):ti,ab,kw or (TUG):ti,ab,kw or (Berg Balance Scale):ti,ab,kw or (BBS):ti,ab,kw **46432**<br>**#11** #9 OR #10 **609633**<br>**#12** (Randomized Controlled Trial):ti,ab,kw or (RCT):ti,ab,kw or (randomised):ti,ab,kw or (randomised):ti,ab,kw or (randomly):ti,ab,kw or (placebo):ti,ab,kw **1520483**<br>**#13** #5 AND #6 AND #11 AND #12 **117**
Embase	**#1** 'aged'/exp **4628533**<br>**#2** 'older adults':ab,ti OR 'old people':ab,ti OR 'elders':ab,ti OR 'senior':ab,ti OR 'geriatric':ab,ti OR 'elderly':ab,ti **782954**<br>**#3** #1 OR #2 **4857444**<br>**#4** 'sensorimotor exercise':ti,ab OR 'sensory motor training':ti,ab OR 'sensorimotor training':ti,ab OR 'somatosensory exercises':ti,ab OR 'proprioceptive exercise':ti,ab OR 'proprioceptive training':ti,ab OR 'proprioceptive neuromuscular facilitation':ti,ab OR 'proprioceptive neuromuscular facilitation technique':ti,ab OR 'proprioceptive neuromuscular facilitation stabilization techniques':ti,ab OR 'pnf':ti,ab **5057**<br>**#5** 'body equilibrium'/exp **28537**<br>**#6** 'proprioception'/exp **19798**<br>**#7** 'postural balance':ab,ti OR 'posture balance':ab,ti OR 'balance, posture':ab,ti OR 'posture balances':ab,ti OR 'posture equilibrium':ab,ti OR 'equilibrium, posture':ab,ti OR 'musculoskeletal equilibrium':ab,ti OR 'posture equilibriums':ab,ti OR 'equilibrium, musculoskeletal':ab,ti OR 'postural equilibrium':ab,ti OR 'equilibrium, postural':ab,ti OR 'balance, postural':ab,ti OR 'postural control':ab,ti OR 'control, postural':ab,ti OR 'postural controls':ab,ti OR 'posture control':ab,ti OR 'control, posture':ab,ti OR 'postural stability':ab,ti OR 'posture controls':ab,ti OR 'postural sway':ab,ti OR 'center of pressure':ab,ti OR 'cop':ab,ti OR 'sway area':ab,ti OR 'sway velocity':ab,ti OR 'sensory organization test':ab,ti OR 'sot':ab,ti OR 'muscle activation':ab,ti OR 'neuromuscular control':ab,ti OR 'tug':ab,ti OR 'timed up and go':ab,ti OR 'berg balance scale':ab,ti OR 'bbs':ab,ti **75821**<br>**#8** #5 OR #6 OR #7 **108261**<br>**#9** 'randomized controlled trial'/exp OR 'randomized controlled trial':ti,ab OR 'randomised':ti,ab OR 'randomly':ti,ab OR 'placebo':ti,ab **1911781**<br>**#10** #3 AND #4 AND #8 AND #9 **127**
Web of Science	TS=("older adults" OR "old people" OR "elders" OR "senior*" OR "geriatric*" OR "elderly" OR "Aged") AND TS=("Sensorimotor Exercise" OR "Sensory Motor Training" OR "Sensorimotor training" OR "somatosensory exercises" OR "Proprioceptive Exercise" OR "Proprioceptive Training" OR "Proprioceptive Neuromuscular Facilitation" OR "Proprioceptive Neuromuscular Facilitation Technique" OR "Proprioceptive Neuromuscular Facilitation Stabilization Techniques" OR "PNF") AND TS=("postural balance" OR "posture balance" OR "balance, posture" OR "posture balances" OR "posture equilibrium" OR "equilibrium,

	posture" OR "musculoskeletal equilibrium" OR "posture equilibriums" OR "equilibrium, musculoskeletal" OR "postural equilibrium" OR "equilibrium, postural" OR "balance, postural" OR "postural control" OR "control, postural" OR "postural controls" OR "posture control" OR "control, posture" OR "posture controls" OR "postural stability" OR "postural sway" OR "center of pressure" OR "cop" OR "sway area" OR "sway velocity" OR "sensory organization test" OR "sot" OR "muscle activation" OR "neuromuscular control" OR "tug" OR "timed up and go" OR "berg balance scale" OR "bbs") AND TS=("Randomized Controlled Trial" OR "randomized controlled trial" OR "randomised" OR "randomly" OR "placebo") **49**
EBSCOhost	**S1** (MH"Aged+") OR TI("older adults" OR "old people" OR" elders" OR "senior*" OR "geriatric*" OR "elderly") OR AB("older adults" OR "old people" OR "elders" OR "senior*" OR "geriatric*" OR "elderly") **6,914,194**<br>**S2** TI("Sensorimotor Exercise" OR "Sensory Motor Training" OR "Sensorimotor training" OR "somatosensory exercises" OR "Proprioceptive Exercise" OR "Proprioceptive Training" OR "Proprioceptive Neuromuscular Facilitation" OR "Proprioceptive Neuromuscular Facilitation Technique" OR "Proprioceptive Neuromuscular Facilitation Stabilization Techniques" OR "PNF") OR AB("Sensorimotor Exercise" OR "Sensory Motor Training" OR "Sensorimotor training" OR "somatosensory exercises" OR "Proprioceptive Exercise" OR "Proprioceptive Training" OR "Proprioceptive Neuromuscular Facilitation" OR "Proprioceptive Neuromuscular Facilitation Technique" OR "Proprioceptive Neuromuscular Facilitation Stabilization Techniques" OR "PNF") **5,347**<br>**S3** (MH "Postural Balance+") OR (MH "Proprioception+") OR TI("balance control" OR "postural stability" OR "postural sway" OR "center of pressure" OR "COP" OR "sway area" OR "sway velocity" OR "sensory organization test" OR "SOT" OR "muscle activation" OR "neuromuscular control" OR "EMG" OR "Electromyography" OR "Timed Up and Go" OR "TUGT" OR "Berg Balance Scale" OR "BBS") OR AB("balance control" OR "postural stability" OR "postural sway" OR "center of pressure" OR "COP" OR "sway area" OR "sway velocity" OR "sensory organization test" OR "SOT" OR "muscle activation" OR "neuromuscular control" OR "EMG" OR "Electromyography" OR "Timed Up and Go" OR "TUGT" OR "Berg Balance Scale" OR "BBS") **225,640**<br>**S4** (MH "Randomized Controlled Trials+") OR TI("randomized controlled trial" OR "randomised" OR "randomly" OR "placebo") OR AB("randomized controlled trial" OR "randomised" OR "randomly" OR "placebo") **1,769,106**<br>**S5** S1 AND S2 AND S3 AND S4 **90**

Appendix B: Study Characteristics

Table A2. Characteristics of the included randomized controlled trials.

Study	Population	Research object			Intervention measure				Outcome
		Gender	Type	Age	Intervention	Control	Duration	Frequency	
Freire (2024) Brazil	EG: 21<br>CG: 24	M/F	N/A	EG: 84.10 ± 7.99<br>CG: 85.13 ± 8.94	ST	Conventional exercises	12 weeks	Three times a week; 30 min	TUGT↑
Jimenez-Mazuelas (2024) Spain	EG: 21<br>CG: 23	M/F	Diabetes	EG/CG: 70 ± 8	ST	Conventional therapy	8 weeks	Twice a week; 60 min	TUGT↑
Shabir (2021) Pakistan	EG: 20<br>CG: 20	F	N/A	EG/CG: 50.27 ± 3.17	ST	Conventional exercises	6 weeks	Three times a week; 20 min	TUGT↑
Sedighi Darijani (2024) Iran	EG: 20<br>CG: 20	M	N/A	EG: 65.15 ± 3.64<br>CG: 65.80 ± 3.48	ST	No training program	5 weeks	Three times a week; 60 min	TUGT↑
Cetinkaya (2025) Turkey	EG: 25<br>CG: 27	M/F	N/A	EG: 69.56 ± 3.48<br>CG: 69.11 ± 3.17	ST (NMT)	Dance therapy	12 weeks	Twice a week; 60 min	BBS↑

Niajalili (2026) Iran	EG: 14<br>CG: 14	M/F	Chronic	EG: 56.29 ± 9.52<br>CG: 59.93 ± 8.96	ST	Conventional therapy	4 weeks	Three times a week; 20 min	TUGT↑
Sadiq (2025) Pakistan	EG: 22<br>CG: 22	M/F	Arthritis	EG: 64.1 ± 5.4;<br>CG: 63.5 ± 5.5	ST	Conventional exercises	12 weeks	Three times a week; 45 min	BBS↑
Ahmad (2019) India	EG: 12<br>CG: 9	M/F	Diabetes	EG:66.75 ± 4.15<br>CG:64.77 ± 4.6	ST	Conventional therapy	8 weeks	Three times a week; 80 min	TUGT↑<br>COP↑
Da Silva (2013) Brazil	EG: 51<br>CG: 51	M/F	Arthritis	EG: 57.90 ± 8.50<br>CG: 58.37 ± 8.11	ST	Conventional therapy	16 weeks	Twice a week; 45 min	TUGT↑<br>BBS↑
Morat (2019) Germany	EG1:15<br>EG2:15<br>CG:15	M/F	N/A	EG1: 67.5 ± 5.1<br>EG2: 69.7 ± 6.2<br>CG: 71.1 ± 5.2	EG1:ST(UN)<br>EG2:ST	No training program	8 weeks	Three times a week; 40 min	TUGT↑<br>COP↑

DEMİR (2022) Turkey	EG: 20<br>CG: 20	M/F	Diabetes	EG:73.50 ± 7.08<br>CG:72.45 ± 7.25	PT	No training program	8 weeks	Three times a week; 45 min	BBS↑
Espejo-Antúnez (2020) Spain	EG: 21<br>CG: 21	M/F	N/A	EG: 83.21 ± 6.59<br>CG: 82.72 ± 6.40	PT	Conventional therapy	12 weeks	Twice a week; 30 min	TUGT↑
Esposito (2021) Italy	EG: 15<br>CG: 15	M/F	N/A	EG/CG:71 ± 4.9	PT	No training program	12 weeks	Two times a week; 60 min	BBS↑
Martínez-Amat (2013) Spain	EG: 20<br>CG: 24	M/F	N/A	EG: 79.35 ± 7.42<br>CG: 77.00 ± 6.90	PT	No training program	12 weeks	Two times a week; 50 min	COP↑
Martínez-López (2014) Spain	EG: 20<br>CG: 24	M/F	N/A	EG: 79.35 ± 7.42<br>CG: 77.0 ± 6.90	PT	Conventional exercises	12 weeks	Two times a week; 50 min	BBS↑
Song (2011) Korea	EG: 19<br>CG: 19	M/F	Diabetes	EG: 72.9 ± 5.6<br>CG: 73.2 ± 5.4	PT	Conventional therapy	8 weeks	Two times a week; 60 min	TUGT↑<br>BBS↑<br>COP↑

Teixeira (2010) Brazil	EG: 43<br>CG: 42	F	Osteopor-osis	EG: 63.1 ± 4.53<br>CG: 62.78 ± 4.87	PT	Conventional therapy	18 weeks	Twice a week; 30 min	TUGT↑<br>BBS↑
Markopo-ulos (2025) Greece	EG: 28<br>CG: 30	M/F	Arthritis	EG:67.50 ± 2.83<br>CG:67.37 ± 3.04	PNF	Conventional exercises	6 weeks	Three times a week; 45 min	TUGT↑<br>BBS↑<br>COP↑
George (2025) India	EG: 16<br>CG: 16	M/F	N/A	EG: 67.88 ± 4.75<br>CG: 66.38 ± 4.15	PNF	Conventional therapy	4 weeks	Three times a week; 30 min	BBS↑
Cellatoğlu (2025) Turkey	EG: 23<br>CG: 25	M/F	Arthritis	EG: 68.48 ± 3.38<br>CG: 69.80 ± 5.08	PNF	Conventional exercises	8 weeks	Three times a week; 60 min	TUGT↑
Kajbafvala (2025) Iran	EG: 26<br>CG: 26	M/F	N/A	EG: 68.31 ± 1.18<br>CG: 70.12 ± 1.49	PNF	Aerobic exercise	4 weeks	Three times a week; 50 min	TUGT↑<br>BBS↑
Mesquita (2015) Brazil	EG: 20<br>CG: 18	F	N/A	EG: 68.5 ± 5.4<br>CG: 71.5 ± 6.2	PNF	No training program	4 weeks	Three times a week; 50 min	TUGT↑<br>BBS↑<br>COP↑

Lamp (2023) Brazil	EG: 25<br>CG: 25	F	N/A	EG/CG:＞70	EG1:PNF(RS)<br>EG2:PNF(SR)	Conventional exercises (Balance training)	N/A	N/A	TUGT↑
Kim (2015) Republic of Korea	EG: 24<br>CG: 24	M/F	Chronic	EG/CG:＞70	PNF	Swiss ball training	6 weeks	Three times a week; 30 min	TUGT↑<br>COP↑

Abbreviations: CG, control group; EG, experimental group; M, male; F, female; N/A, not applicable; ST, sensorimotor training; PT, proprioceptive training; PNF, proprioceptive neuromuscular facilitation; NMT, neuromuscular training; UN, unstable; RS, rhythmic stabilization; SR, stabilizers reversal; TUGT, timed up and go test; BBS, berg balance scale; COP, center of pressure; ↑, Significant Between-Group Improvement.

Appendix C: Overall Meta-analysis Results

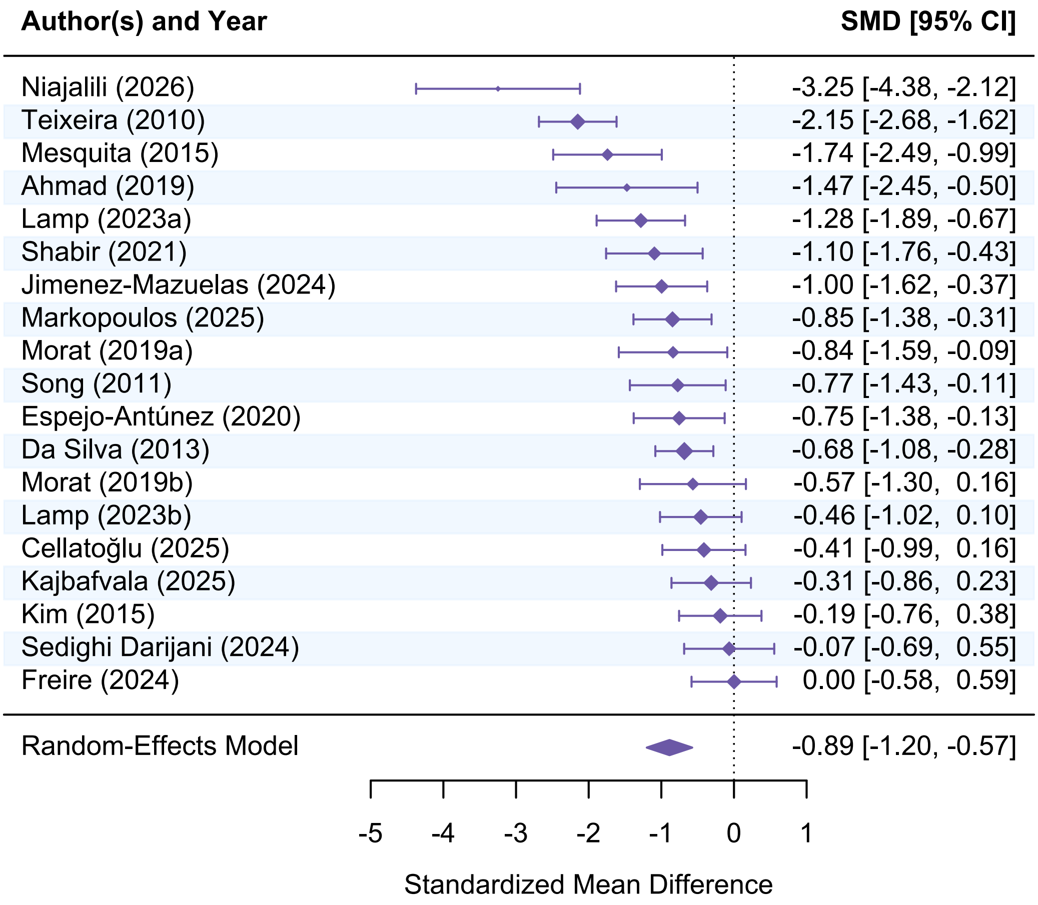

Fig A1. Forest plot of the overall effect on TUGT.

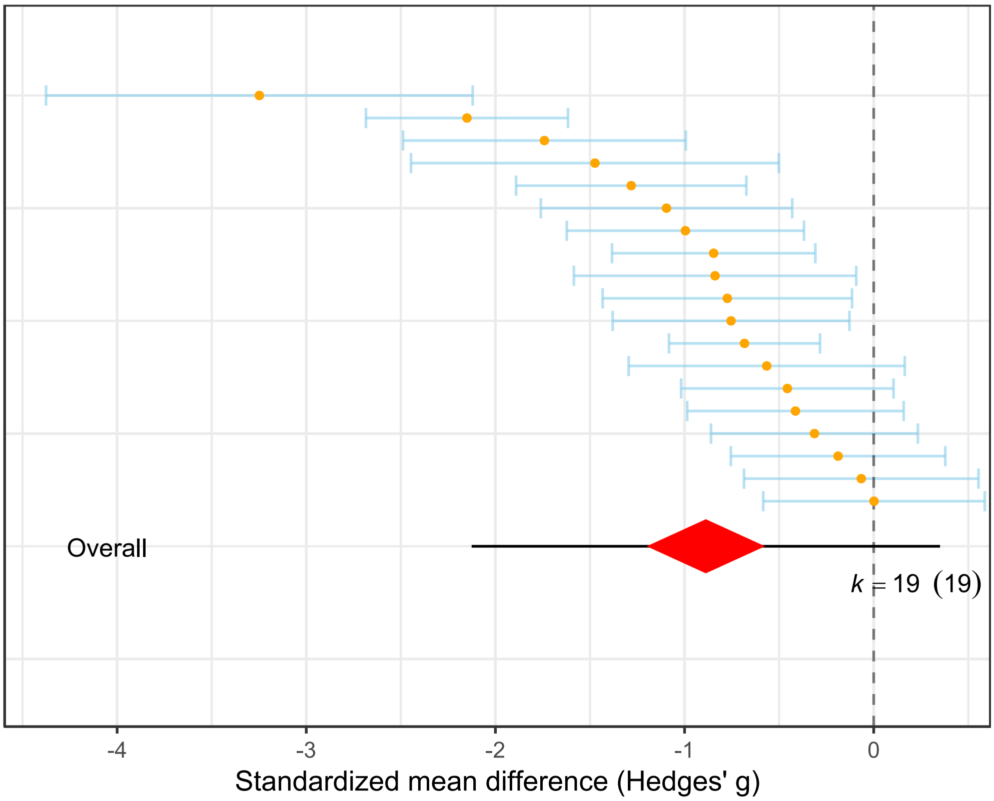

Fig A2. Caterpillar plot of the overall effect on TUGT.

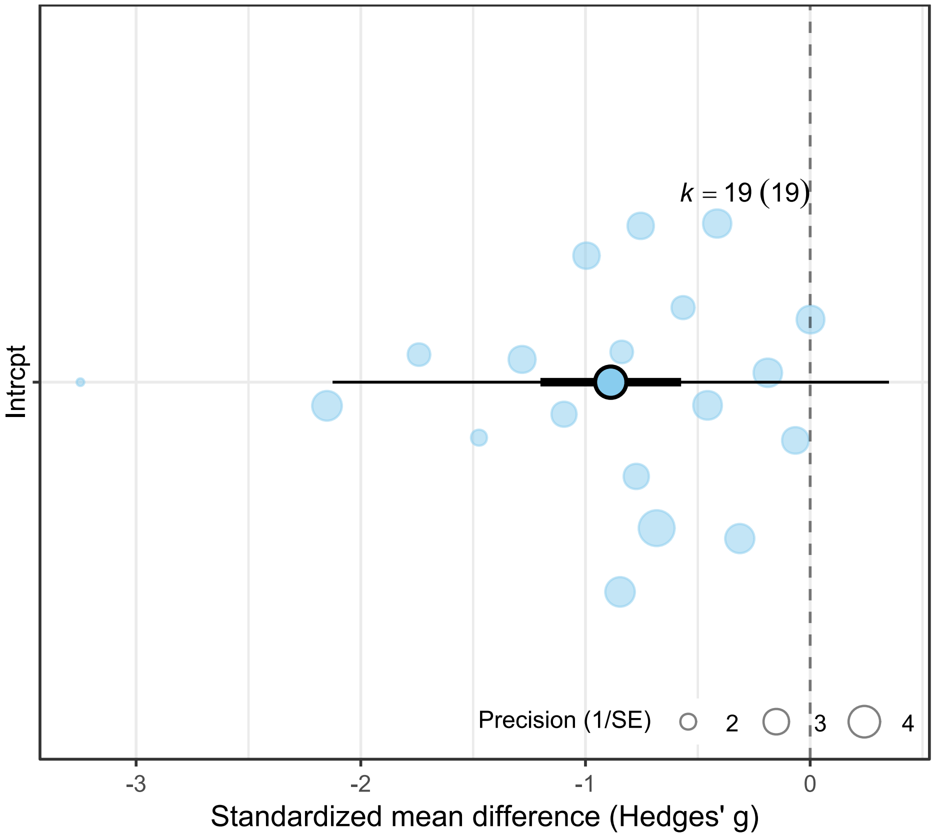

Fig A3. Orchard plot of the overall effect on TUGT.

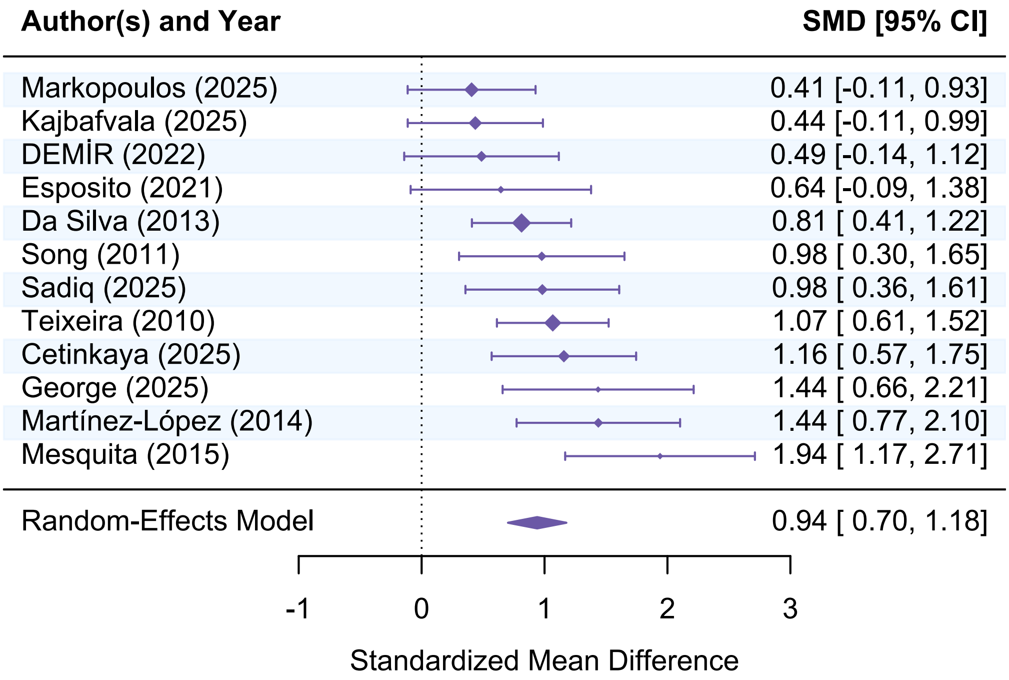

Fig A4. Forest plot of the overall effect on BBS.

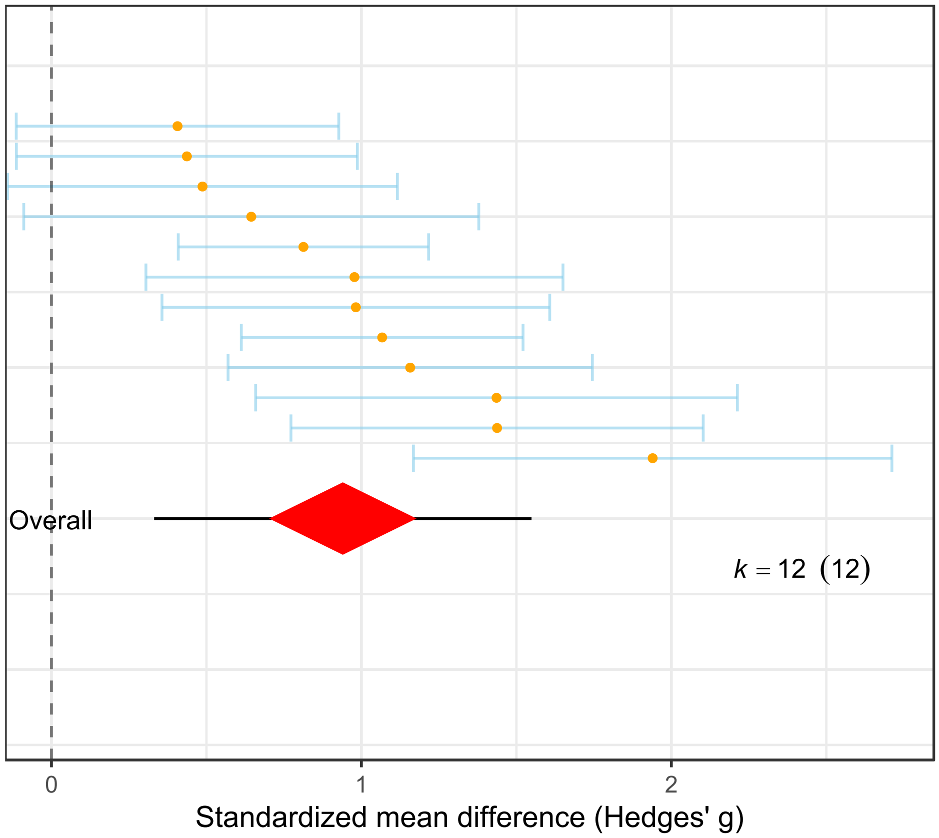

Fig A5. Caterpillar plot of the overall effect on BBS.

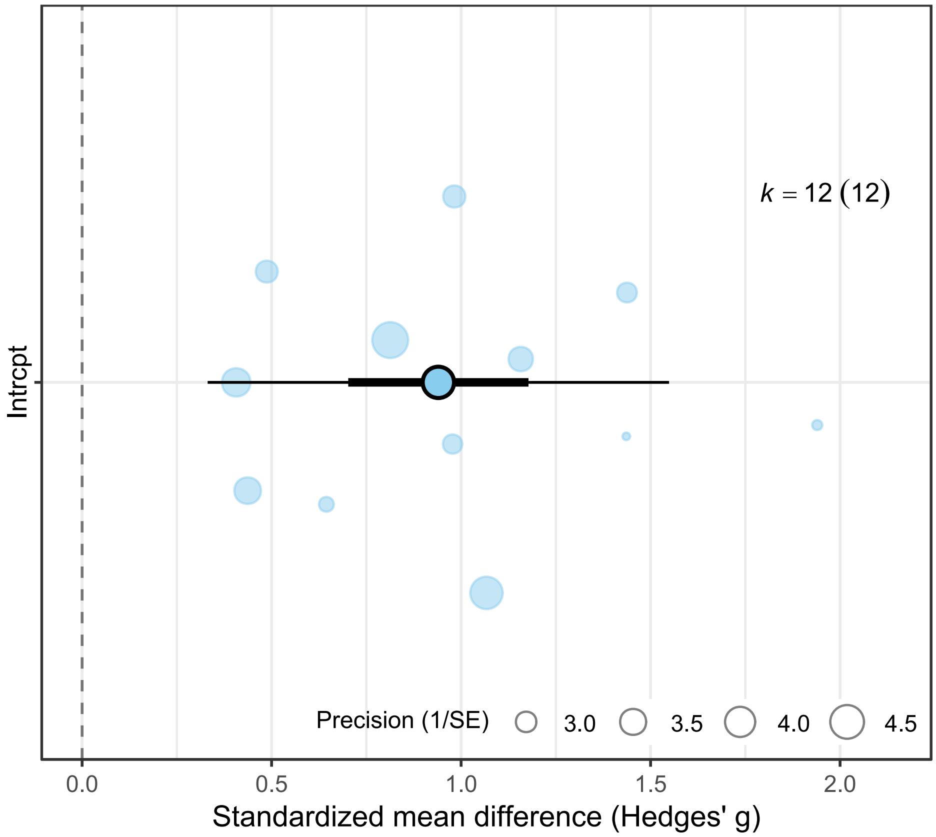

Fig A6. Orchard plot of the overall effect on BBS.

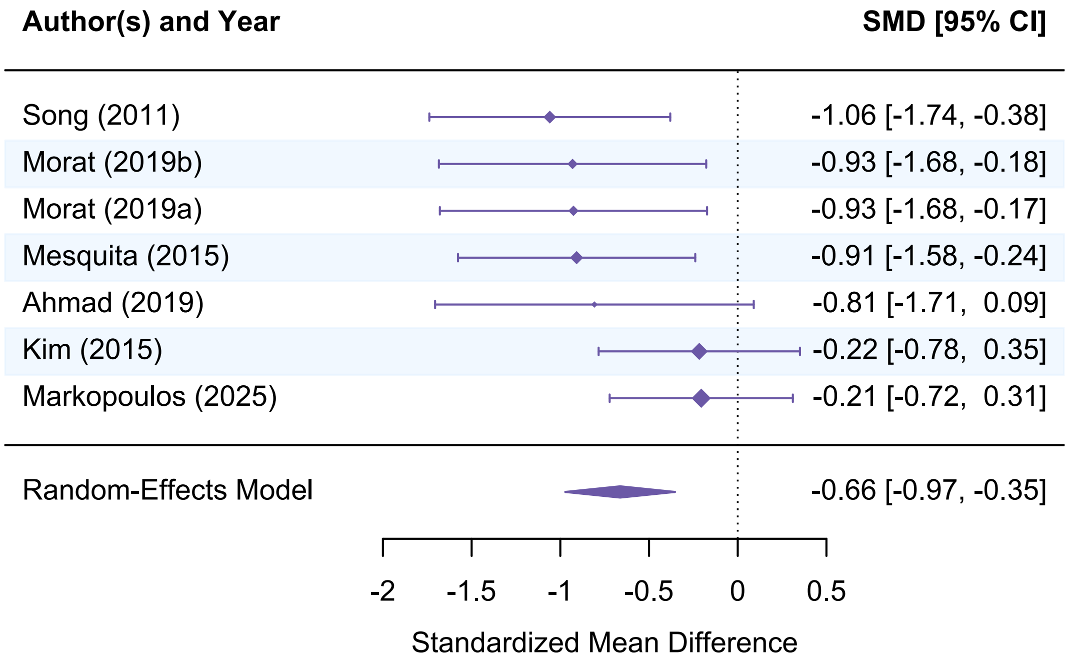

Fig A7. Forest plot of the overall effect on COP-EO.

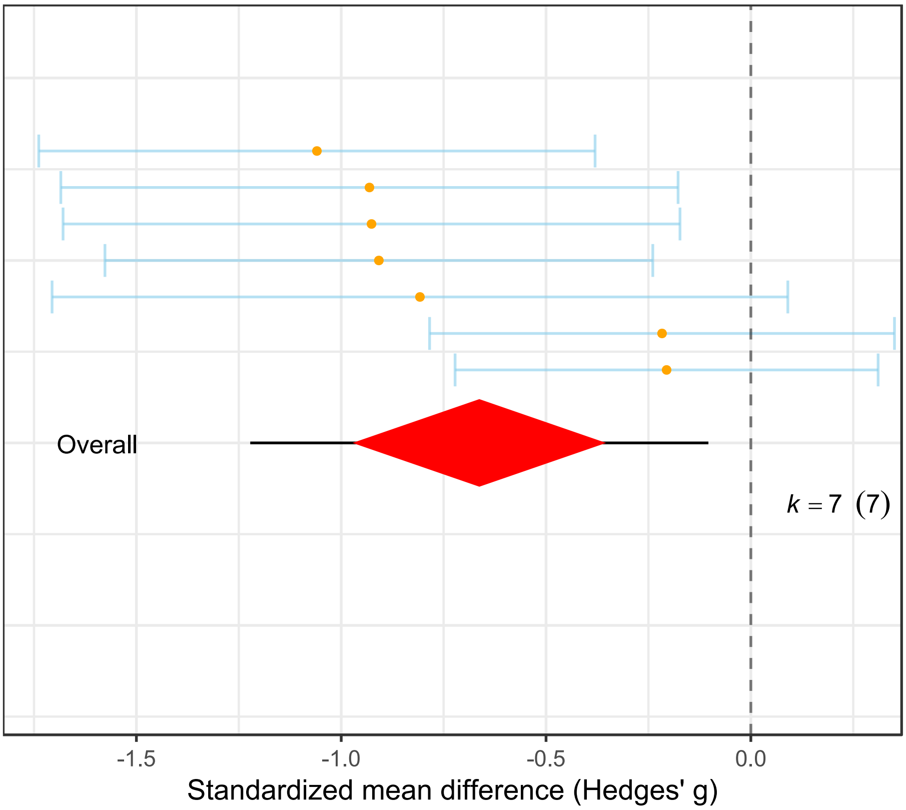

Fig A8. Caterpillar plot of the overall effect on COP-EO.

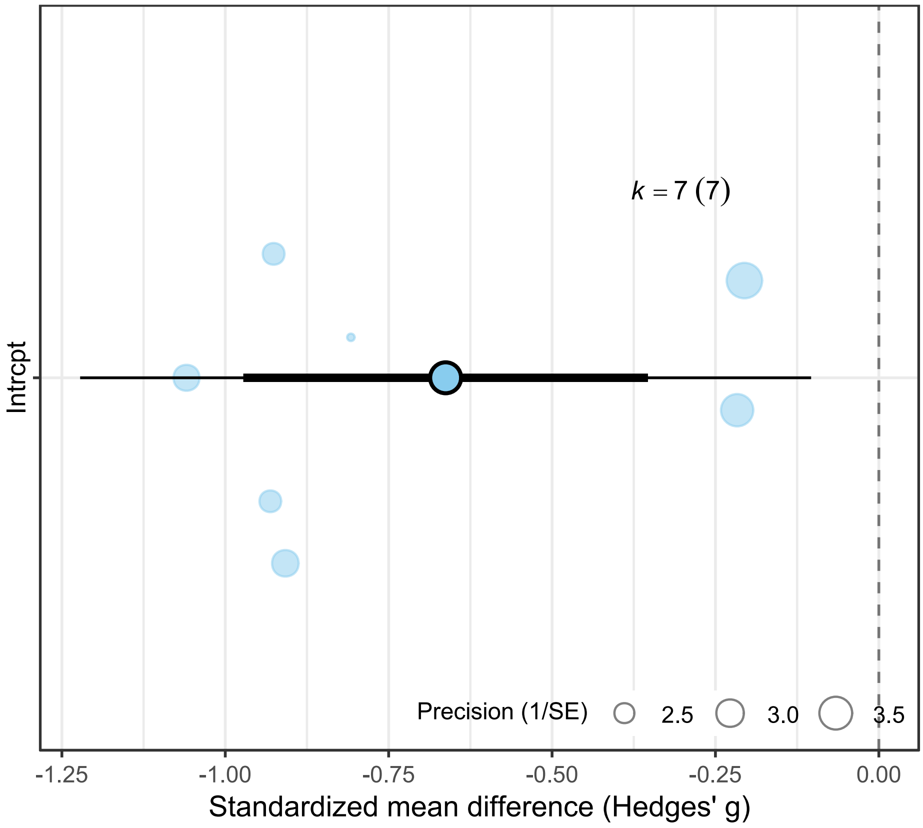

Fig A9. Orchard plot of the overall effect on COP-EO.

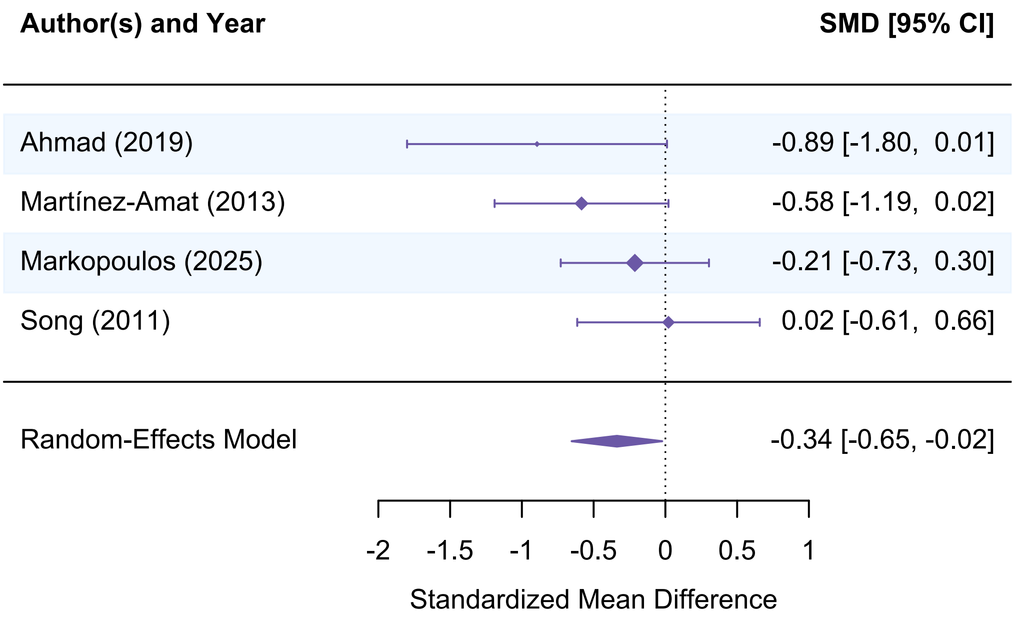

Fig A10. Forest plot of the overall effect on COP-EC.

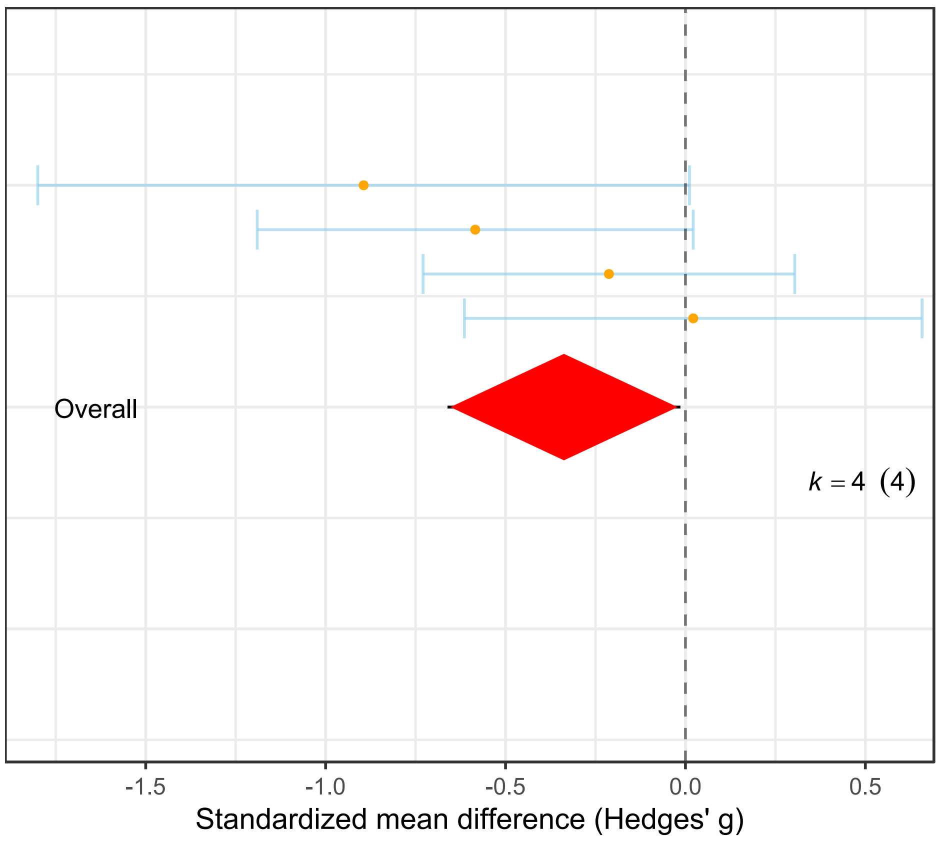

Fig A11. Caterpillar plot of the overall effect on COP-EC.

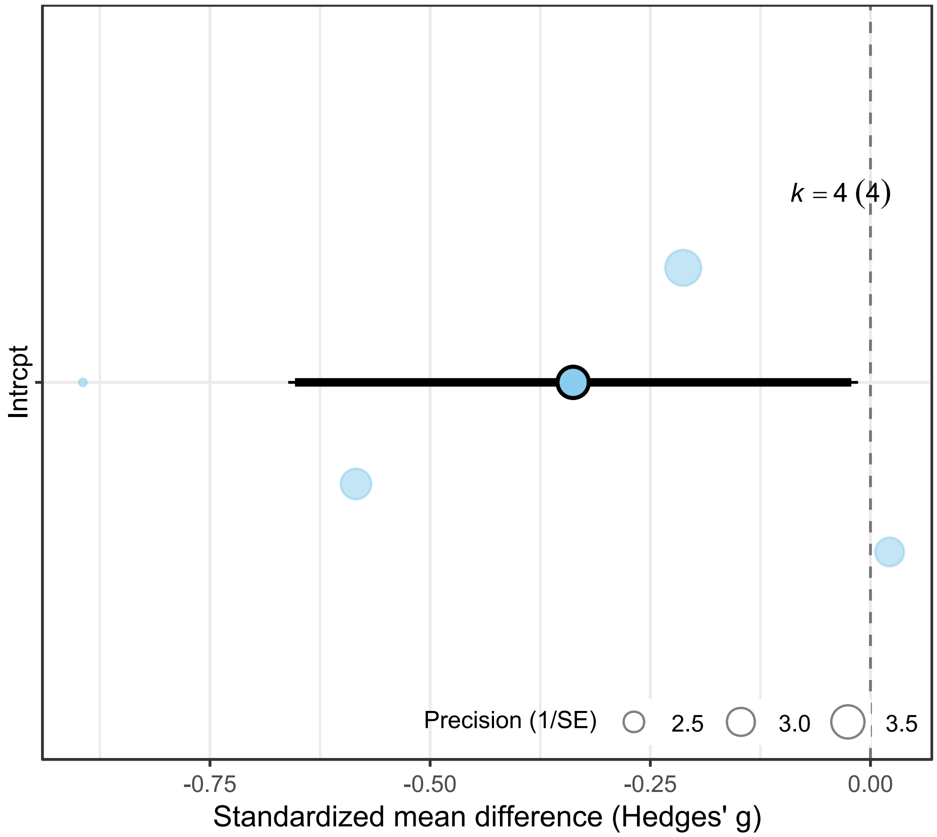

Fig A12. Orchard plot of the overall effect on COP-EC.

Appendix D: Subgroup Analysis

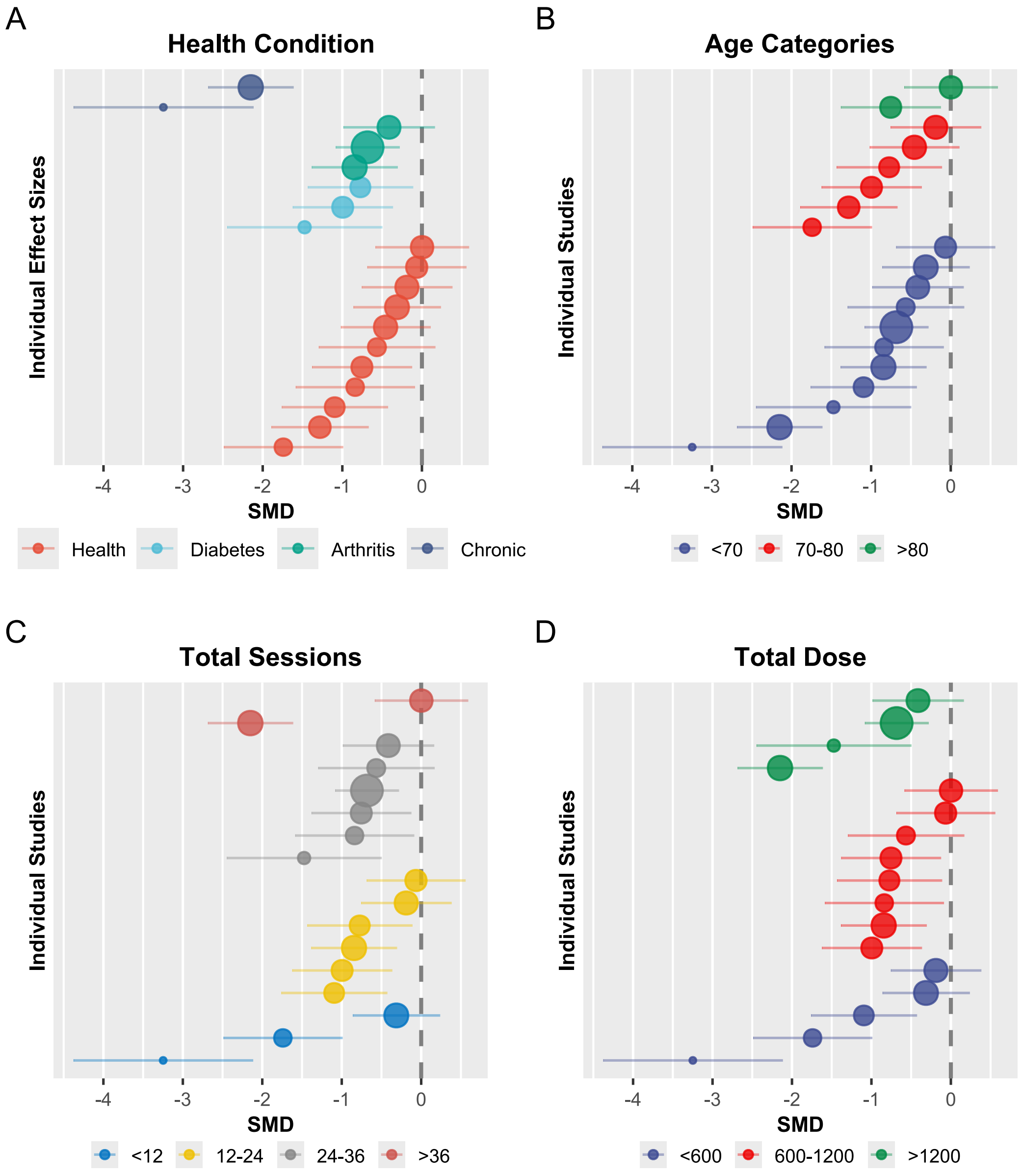

Fig A13. Caterpillar plots for subgroup analyses of TUGT performance categorized by (A) health condition, (B) age categories, (C) total sessions, and (D) total dose.

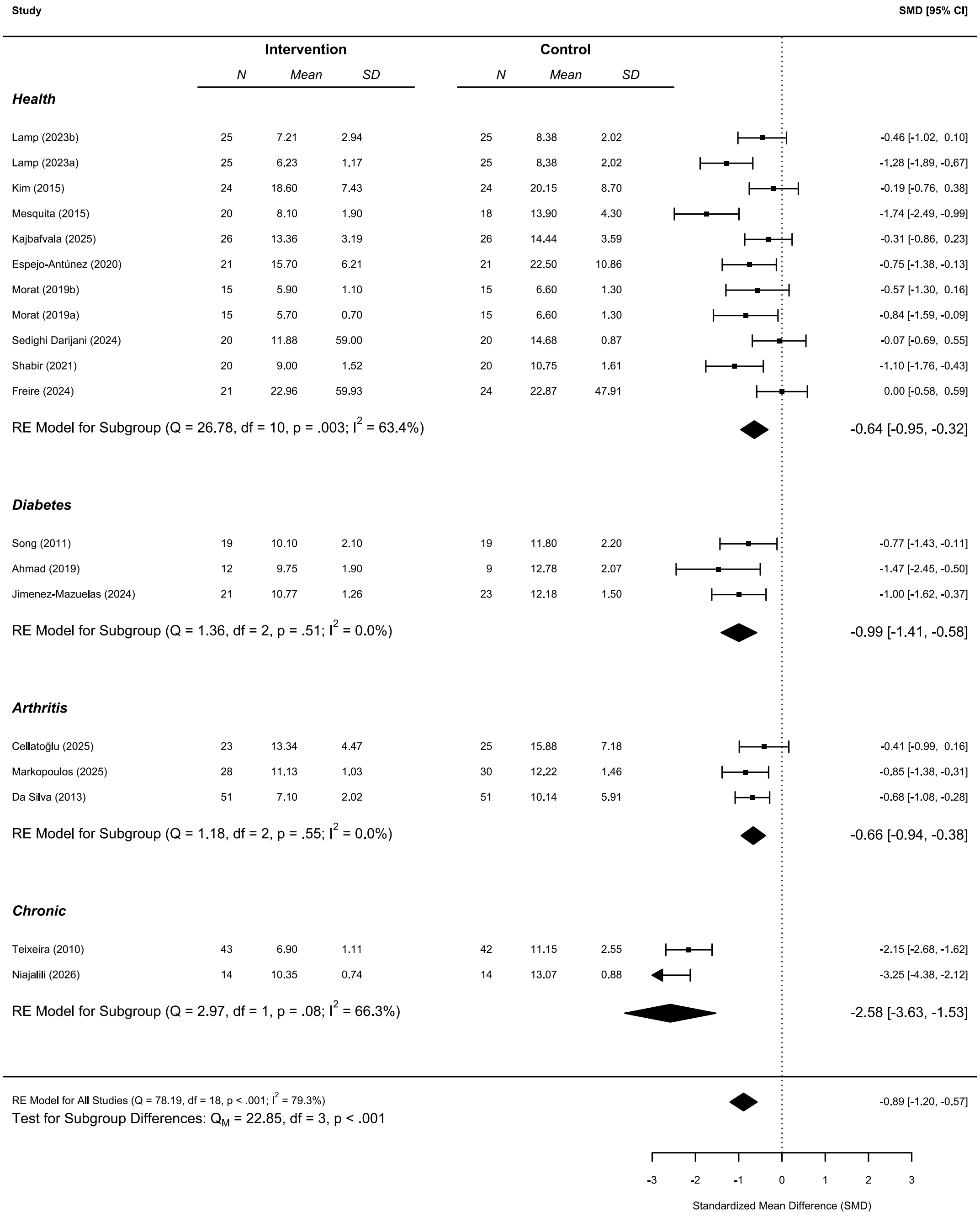

Fig A14. Forest plot for TUGT: Health Condition subgroup.

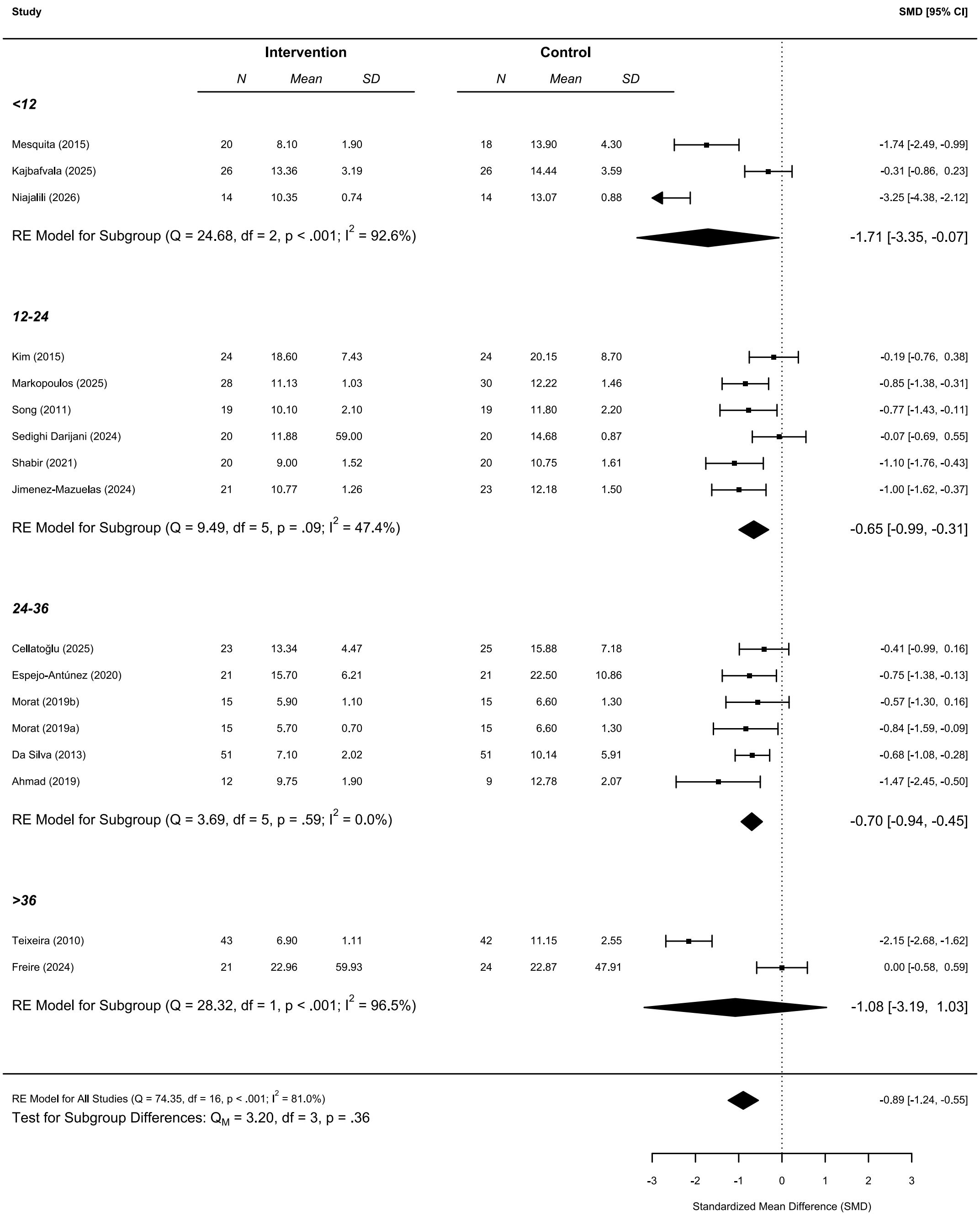

Fig A15. Forest plot for TUGT: Intervention Sessions subgroup.

Study	Intervention N	Intervention Mean	Intervention SD	Control N	Control Mean	Control SD	SMD [95% CI]
<600							
Kim (2015)	24	18.60	7.43	24	20.15	8.70	-0.19 [-0.76, 0.38]
Mesquita (2015)	20	8.10	1.90	18	13.90	4.30	-1.74 [-2.49, -0.99]
Kajbafvala (2025)	26	13.36	3.19	26	14.44	3.59	-0.31 [-0.86, 0.23]
Niajalili (2026)	14	10.35	0.74	14	13.07	0.88	-3.25 [-4.38, -2.12]
Shabir (2021)	20	9.00	1.52	20	10.75	1.61	-1.10 [-1.76, -0.43]
RE Model for Subgroup (Q = 32.26, df = 4, p < .001; I^2 = 91.2%)							-1.25 [-2.29, -0.22]
600-1200							
Markopoulos (2025)	28	11.13	1.03	30	12.22	1.46	-0.85 [-1.38, -0.31]
Song (2011)	19	10.10	2.10	19	11.80	2.20	-0.77 [-1.43, -0.11]
Espejo-Antúnez (2020)	21	15.70	6.21	21	22.50	10.86	-0.75 [-1.38, -0.13]
Morat (2019b)	15	5.90	1.10	15	6.60	1.30	-0.57 [-1.30, 0.16]
Morat (2019a)	15	5.70	0.70	15	6.60	1.30	-0.84 [-1.59, -0.09]
Sedighi Darijani (2024)	20	11.88	59.00	20	14.68	0.87	-0.07 [-0.69, 0.55]
Jimenez-Mazuelas (2024)	21	10.77	1.26	23	12.18	1.50	-1.00 [-1.62, -0.37]
Freire (2024)	21	22.96	59.93	24	22.87	47.91	0.00 [-0.58, 0.59]
RE Model for Subgroup (Q = 10.14, df = 7, p = .18; I^2 = 33.2%)							-0.60 [-0.87, -0.32]
>1200							
Cellatoğlu (2025)	23	13.34	4.47	25	15.88	7.18	-0.41 [-0.99, 0.16]
Teixeira (2010)	43	6.90	1.11	42	11.15	2.55	-2.15 [-2.68, -1.62]
Da Silva (2013)	51	7.10	2.02	51	10.14	5.91	-0.68 [-1.08, -0.28]
Ahmad (2019)	12	9.75	1.90	9	12.78	2.07	-1.47 [-2.45, -0.50]
RE Model for Subgroup (Q = 25.02, df = 3, p < .001; I^2 = 87.2%)							-1.16 [-1.96, -0.36]
RE Model for All Studies (Q = 74.35, df = 16, p < .001; I^2 = 81.0%)							-0.89 [-1.24, -0.55]

Test for Subgroup Differences: Q_M = 2.45, df = 2, p = .29

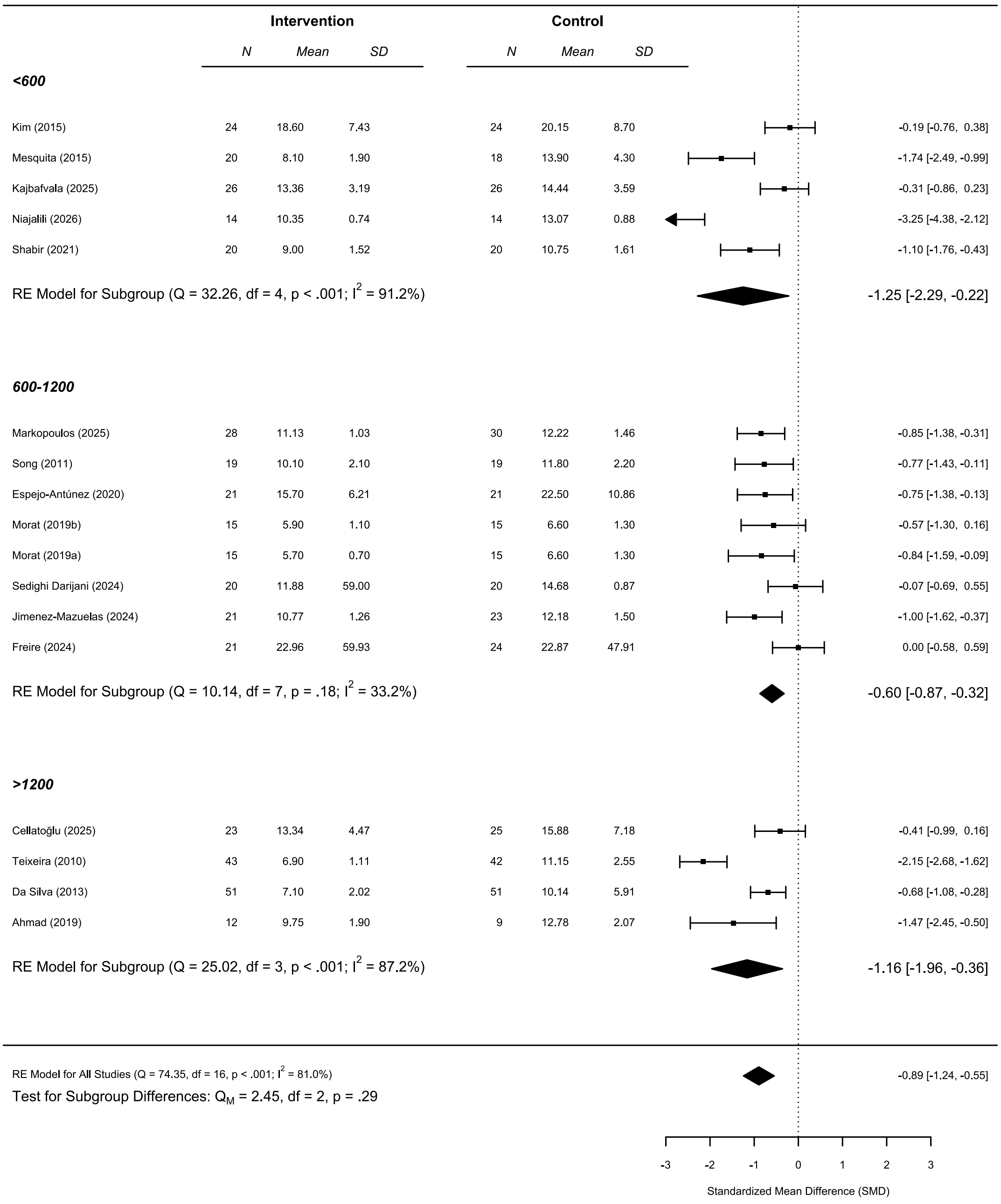

Fig A16. Forest plot for TUGT: Total Dose subgroup.

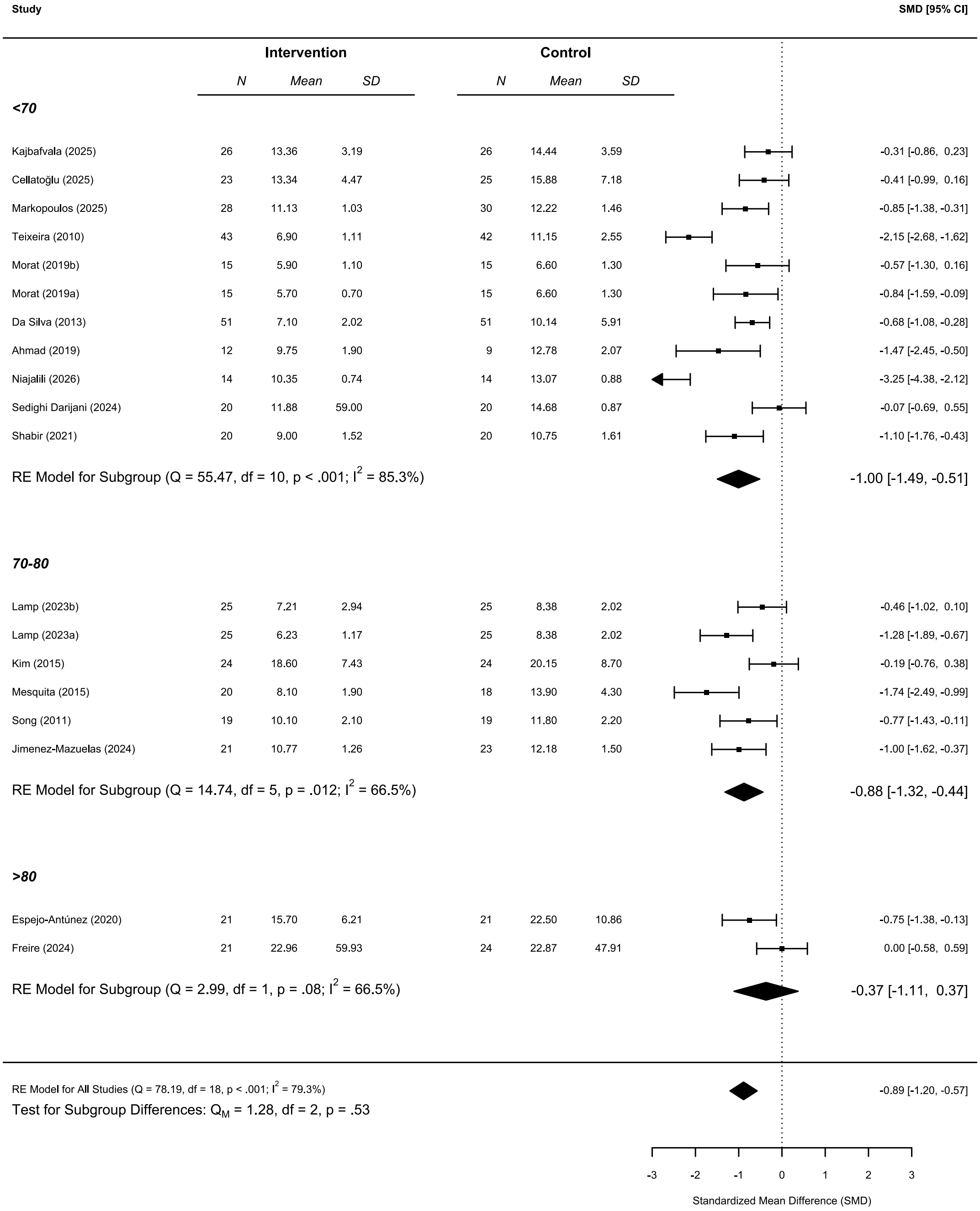

Fig A17. Forest plot for TUGT: Age Categories subgroup.

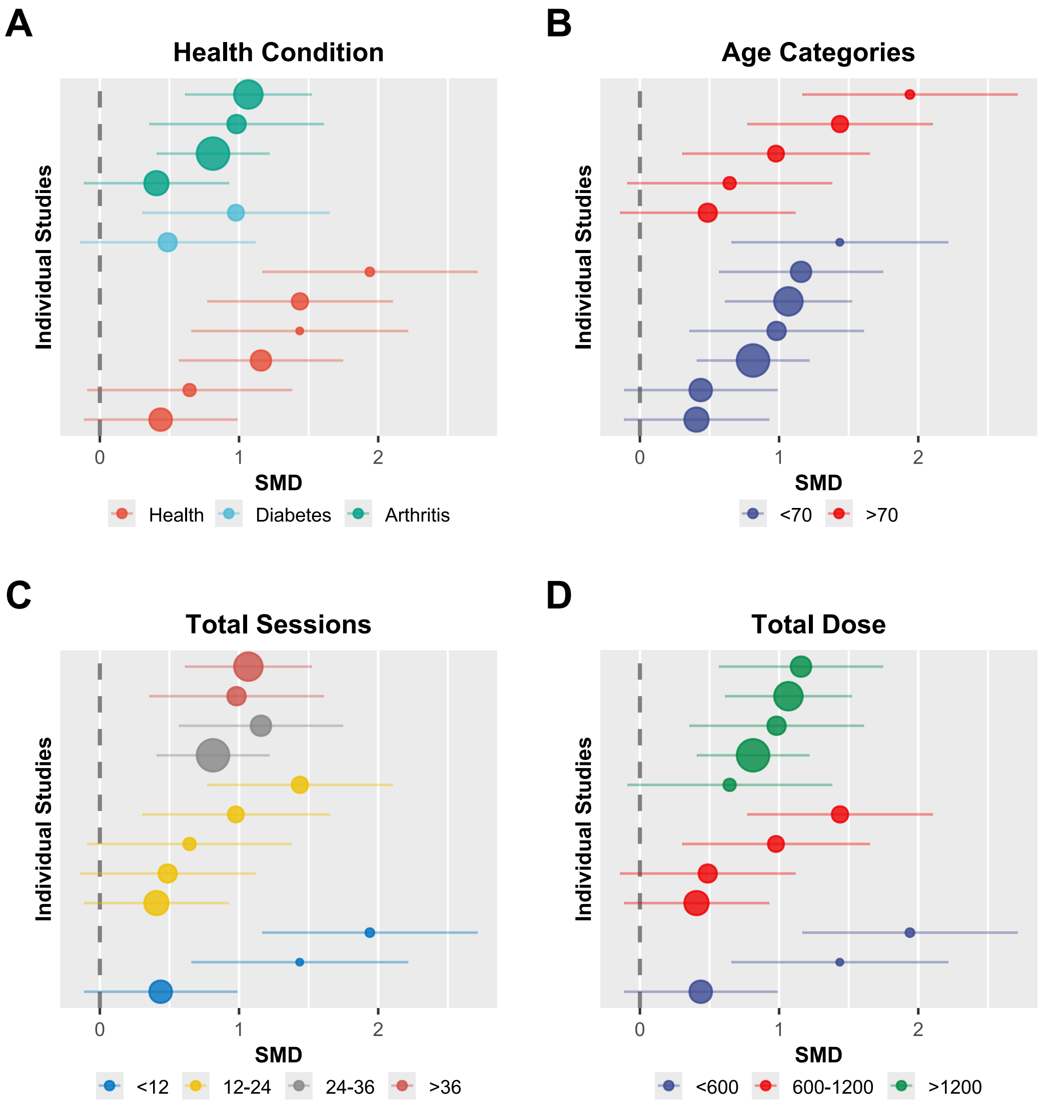

Fig A18. Caterpillar plots for subgroup analyses of BBS performance categorized by (A) health condition, (B) age categories, (C) total sessions, and (D) total dose.

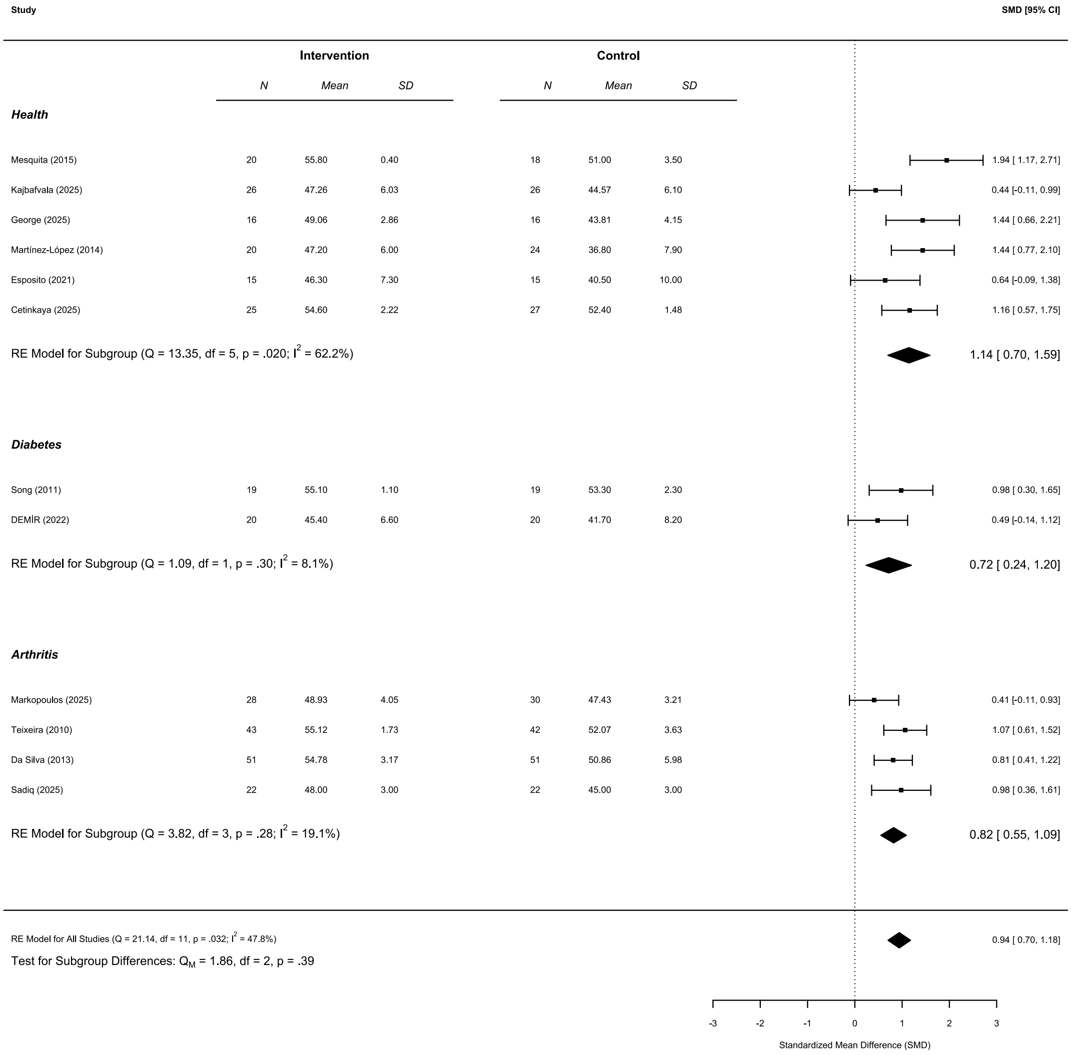

Fig A19. Forest plot for BBS: Health Condition subgroup.

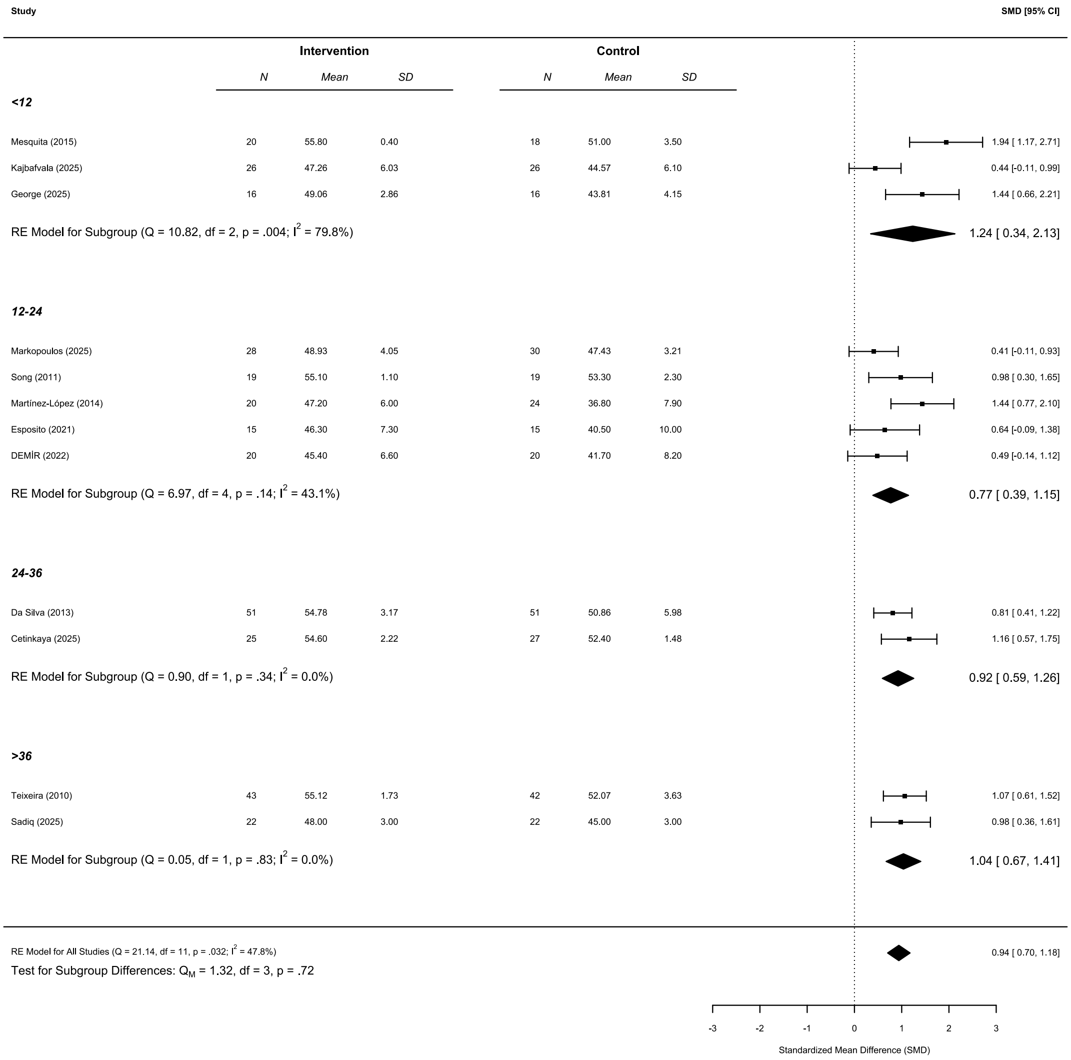

Fig A20. Forest plot for BBS: Intervention Sessions subgroup.

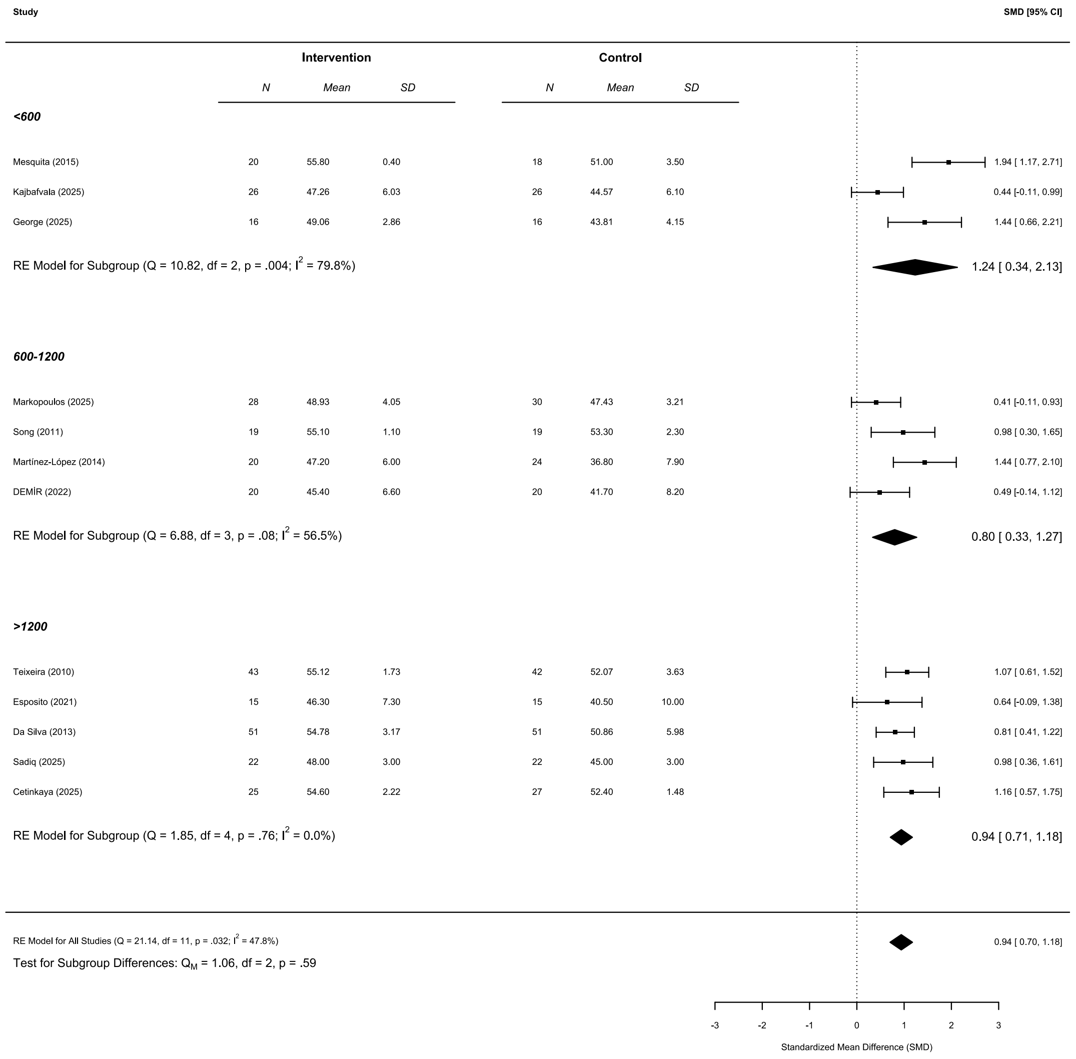

Fig A21. Forest plot for BBS: Total Dose subgroup.

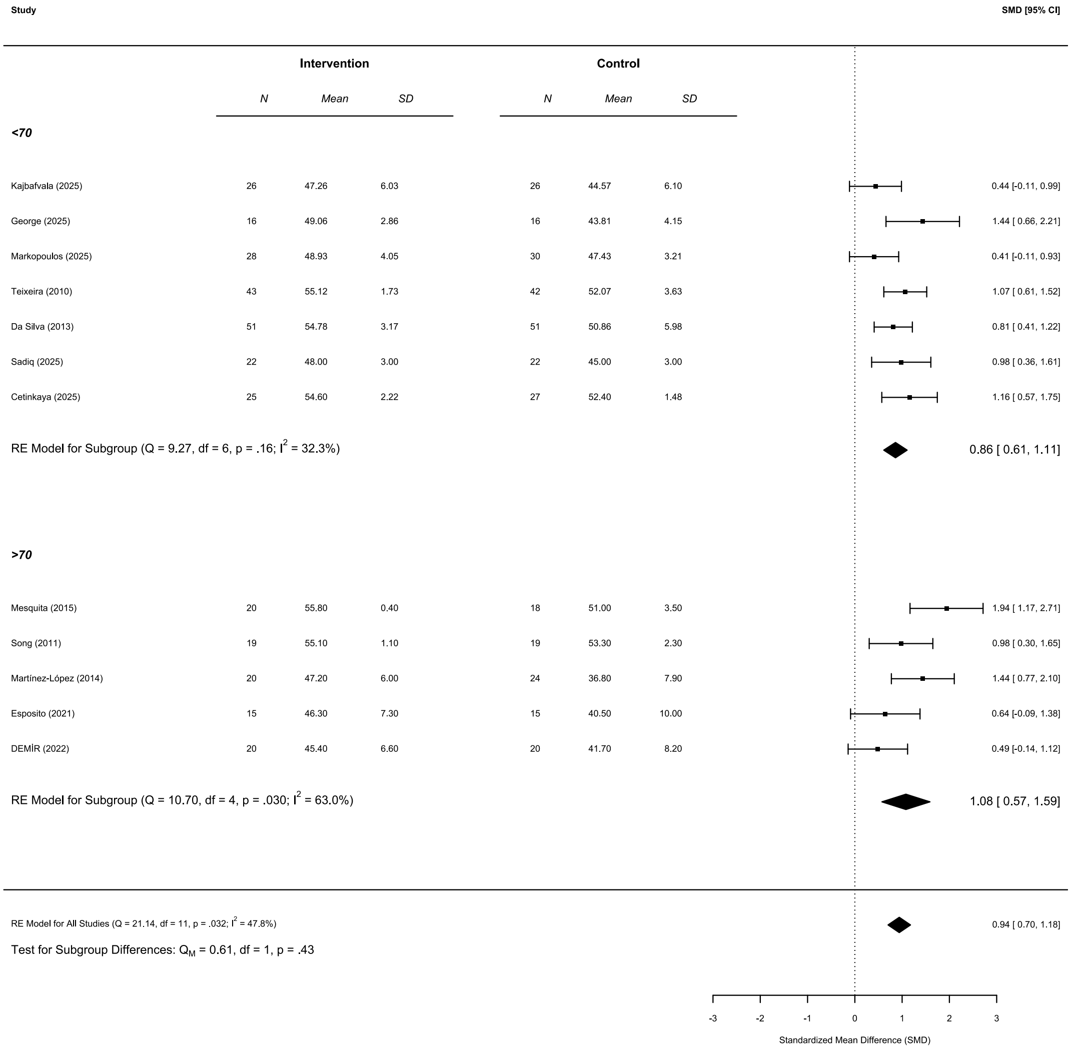

Fig A22. Forest plot for BBS: Age Categories subgroup.

Table A3. Subgroup analysis results for Dynamic Balance (TUGT).

Moderator	Group	*K*	EG(*n*)	CG(*n*)	Hedges' *g* [95%CI]	*Q*	*P*	I^2
Health	Health	11	232	233	-0.64 [-0.95, -0.32]	22.85	***	63.40%
	Diabetes	3	52	51	-0.99 [-1.41, -0.58]			0.00%
	Arthritis	3	102	106	-0.66 [-0.94, -0.38]			0.00%
	Chronic	2	57	56	-2.58 [-3.63, -1.53]			66.30%
Total Sessions	<12	3	60	58	-1.71 [-3.35, -0.07]	3.20	0.36	92.60%
	12-24	6	132	137	-0.65 [-0.99, -0.31]			47.40%
	24-36	6	137	136	-0.70 [-0.94, -0.45]			0.00%
	>36	2	64	66	-1.08 [-3.19, 1.03]			96.50%
Total dose	<600	5	104	102	-1.25 [-2.29, -0.22]	2.45	0.29	91.20%
	600-1200	8	160	167	-0.60 [-0.87, -0.32]			33.20%
	>1200	4	129	127	-1.16 [-1.96, -0.36]			87.20%
Age	<70	11	267	267	-1.00 [-1.49, -0.51]	1.28	0.53	85.30%
	70-80	6	134	134	-0.88 [-1.32, -0.44]			66.50%
	>80	2	42	45	-0.37 [-1.11, 0.37]			66.50%

Note: *K*, number of independent comparisons; EG(*n*), number of participants in the experimental group; CG(*n*), number of participants in the control group; CI, confidence interval; *Q*, Cochran's *Q* statistic for between-subgroup heterogeneity; *P*, probability value for the *Q* test; I^2, Higgins' statistic for within-subgroup heterogeneity. ***$p < 0.001$.

Table A4. Subgroup analysis results for Static Balance (BBS).

Moderator	Group	*K*	EG(*n*)	CG(*n*)	Hedges' *g* [95%CI]	*Q*	*P*	I^2
Health	Health	6	122	126	1.14 [0.70, 1.59]	1.86	0.40	62.20%
	Diabetes	2	39	39	0.72 [0.24, 1.20]			8.10%
	Arthritis	4	144	145	0.82 [0.55, 1.09]			19.10%
Total Sessions	<12	3	62	60	1.24 [0.34, 2.13]	1.32	0.72	79.80%
	12-24	5	102	108	0.77 [0.39, 1.15]			43.10%
	24-36	2	76	78	0.92 [0.59, 1.26]			0.00%
	>36	2	65	64	1.04 [0.67, 1.41]			0.00%
Total dose	<600	3	62	60	1.24 [0.34, 2.13]	1.06	0.59	79.80%
	600-1200	4	87	93	0.80 [0.33, 1.27]			56.50%
	>1200	5	156	157	0.94 [0.71, 1.18]			0.00%
Age	<70	7	211	214	0.86 [0.61, 1.11]	0.61	0.43	32.30%
	>70	5	94	96	1.08 [0.57, 1.59]			63.00%

Note: *K*, number of independent comparisons; EG(*n*), number of participants in the experimental group; CG(*n*), number of participants in the control group; CI, confidence interval; *Q*, Cochran's *Q* statistic for between-subgroup heterogeneity; *P*, probability value for the *Q* test; I^2, Higgins' statistic for within-subgroup heterogeneity.

Appendix E: Meta-regression Analysis

Table A5. Non-linear Dose-response meta-regression results of TUGT.

	β	*SE*	*Z*-value	*P*-value	*CI.lb*	*CI.ub*
Intercept	-2.478	0.667	-3.713	0.001	-3.786	-1.170
rcs1	0.003	0.001	2.682	0.007	0.001	0.004
rcs2	-0.003	0.001	-2.920	0.004	-0.005	-0.001
Model Fit Statistics						
R^2	32.90%					
Q	$Q_M(df = 2) = 8.53$*					
$P_{\text{non-linearity}}$	0.004					

Note: *β*, regression coefficient; *SE*, standard error; *Z*, z-test statistic; *P*, probability value; *CI.lb*, lower bound of 95% confidence interval; *CI.ub*, upper bound of 95% confidence interval; R^2, amount of heterogeneity accounted for; Q, test of moderators. * $p < 0.05$.

Table A6. Non-linear Dose-response meta-regression results of BBS.

	β	*SE*	*Z*-value	*P*-value	*CI.lb*	*CI.ub*
Intercept	1.529	0.652	2.344	0.019	0.251	2.808
rcs1	-0.001	0.001	-0.974	0.330	-0.003	0.001
rcs2	0.001	0.001	0.969	0.333	-0.001	0.003
Model Fit Statistics						
R^2	0.00%					
Q	$Q_M(df = 2) = 0.981$					
$P_{\text{non-linearity}}$	0.333					

Note: *β*, regression coefficient; *SE*, standard error; *Z*, z-test statistic; *P*, probability value; *CI.lb*, lower bound of 95% confidence interval; *CI.ub*, upper bound of 95% confidence interval; R^2, amount of heterogeneity accounted for; Q, test of moderators.

Table A7. Linear meta-regression results of baseline age on TUGT.

	β	*SE*	*Z*-value	*P*-value	*CI.lb*	*CI.ub*
Intercept	-3.532	1.345	-2.627	0.009	-6.167	-0.897
Age (years)	0.039	0.020	1.983	0.047	0.001	0.078
Model Fit Statistics						
R^2	14.77%					
Q	$Q_M(df = 1) = 3.933$*					
P	0.047					

Note: *β*, regression coefficient; *SE*, standard error; *Z*, z-test statistic; *P*, probability value; *CI.lb*, lower bound of 95% confidence interval; *CI.ub*, upper bound of 95% confidence interval; R^2, amount of heterogeneity accounted for; Q, test of moderators. * $p < 0.05$.

Table A8. Linear meta-regression results of baseline age on BBS.

	β	*SE*	*Z*-value	*P*-value	*CI.lb*	*CI.ub*
Intercept	0.230	1.588	0.145	0.885	-2.882	3.342
Age (years)	0.011	0.023	0.451	0.652	-0.035	0.056
Model Fit Statistics						
R^2	0.00%					
Q	$Q_M(df = 1) = 0.203$					
P	0.652					

Note: *β*, regression coefficient; *SE*, standard error; *Z*, z-test statistic; *P*, probability value; *CI.lb*, lower bound of 95% confidence interval; *CI.ub*, upper bound of 95% confidence interval; R^2, amount of heterogeneity accounted for; Q, test of moderators.

Appendix F: Publication Bias and Sensitivity Analysis

Table A9. Summary of publication bias (Egger's test) and sensitivity analysis.

Outcomes	Hedges's *g*	*CI.lb*	*CI.ub*	*Eggers Test*	*Trim and Fill*	*Sensitivity analysis*
TUGT	-0.612	-1.007	-0.217	0.005	Yes	Stable
BBS				0.105	No	Stable
COP-EO	-0.779	-1.103	-0.455	0.024	Yes	Stable
COP-EC				0.259	No	Stable

Note: *CI.lb*, lower bound of 95% confidence interval; *CI.ub*, upper bound of 95% confidence interval;

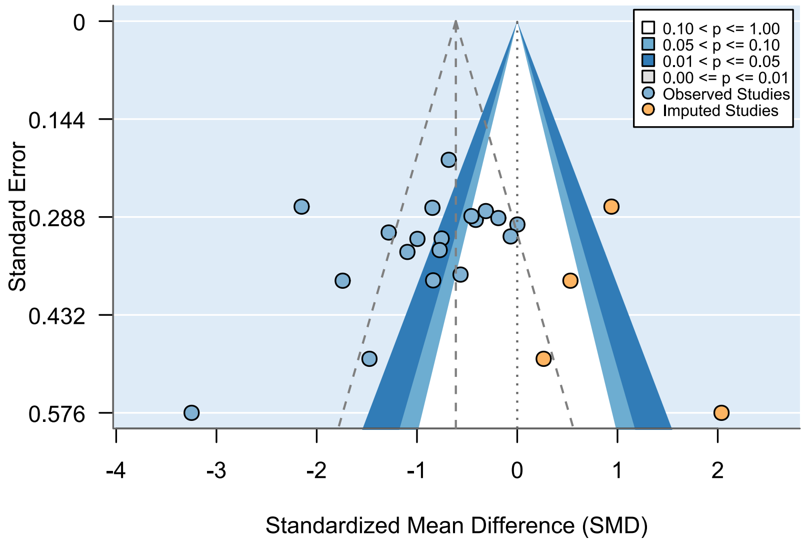

Fig A23. Trim-and-fill funnel plot for TUGT.

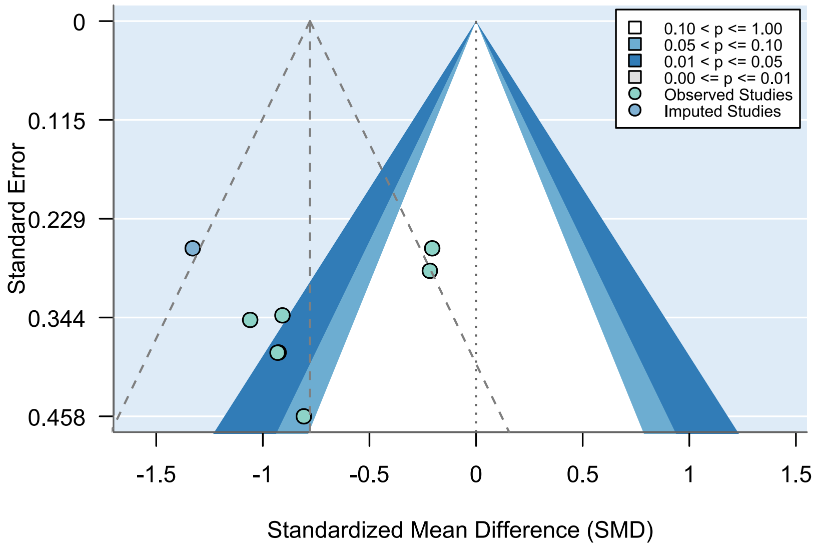

Fig A24. Trim-and-fill funnel plot for COP-EO.

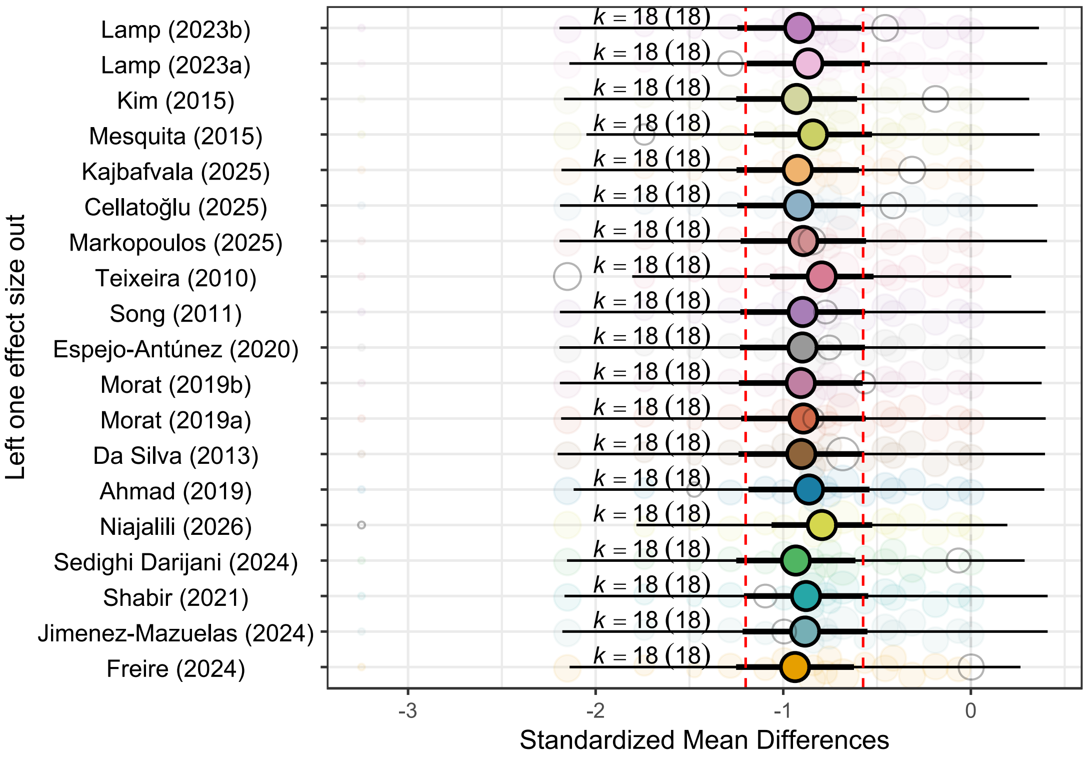

Fig A25. Leave-one-out sensitivity analysis for TUGT.

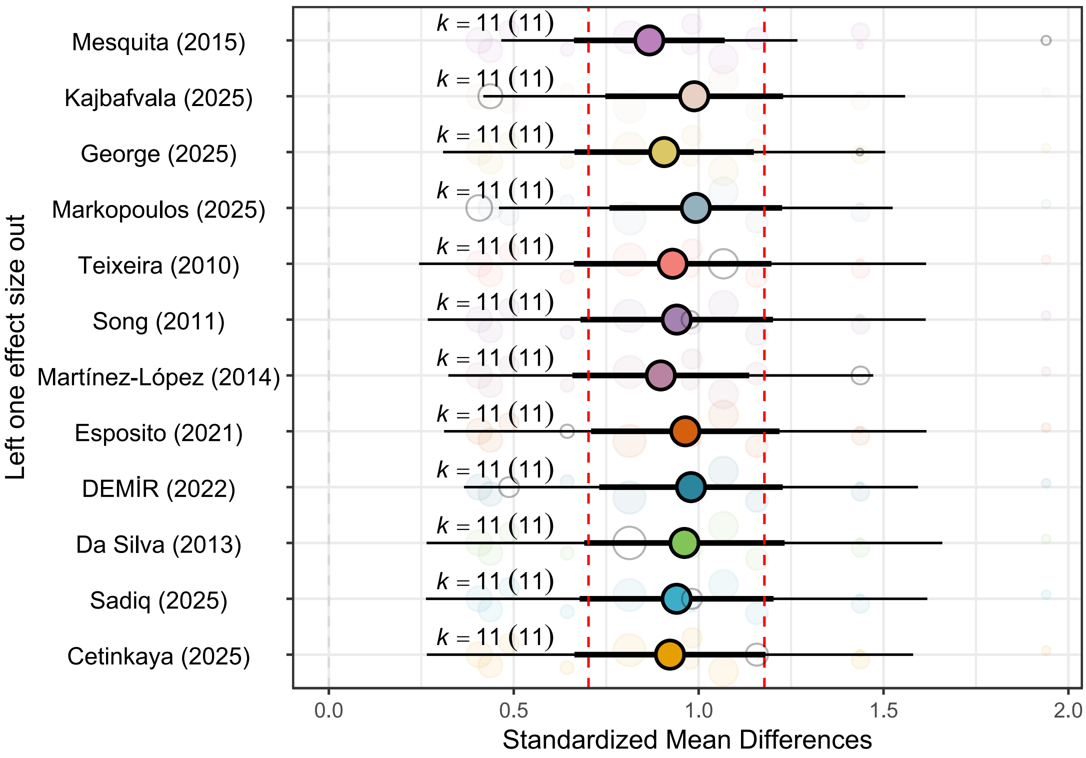

Fig A26. Leave-one-out sensitivity analysis for BBS.

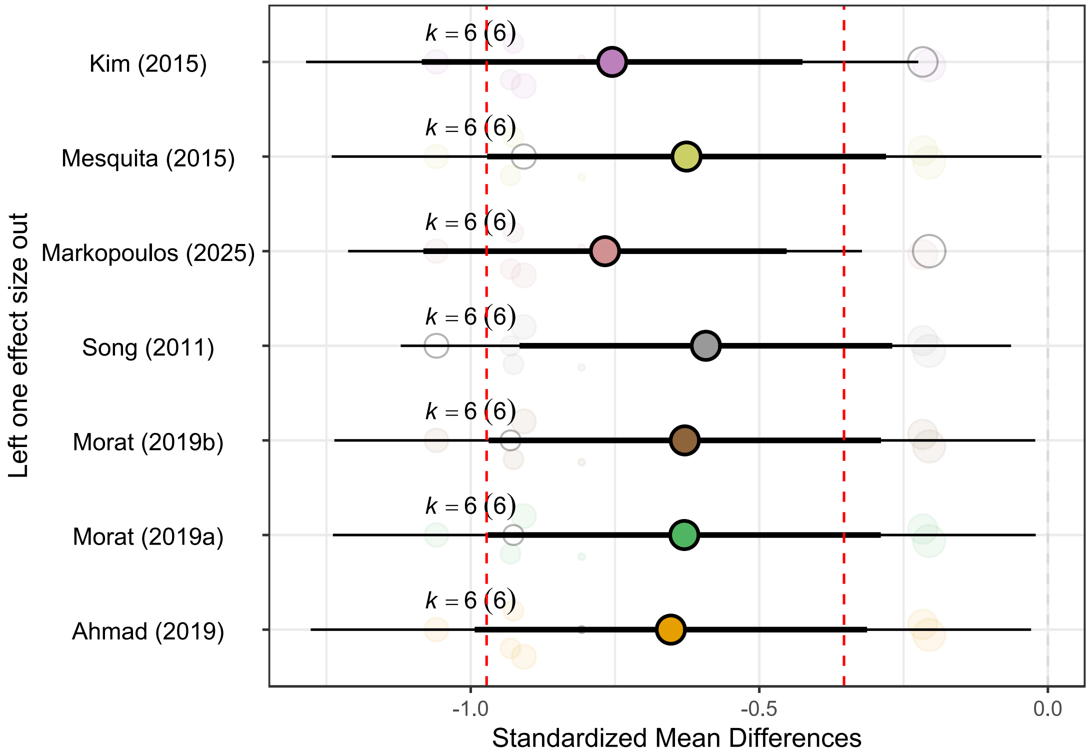

Fig A27. Leave-one-out sensitivity analysis for COP-EO.

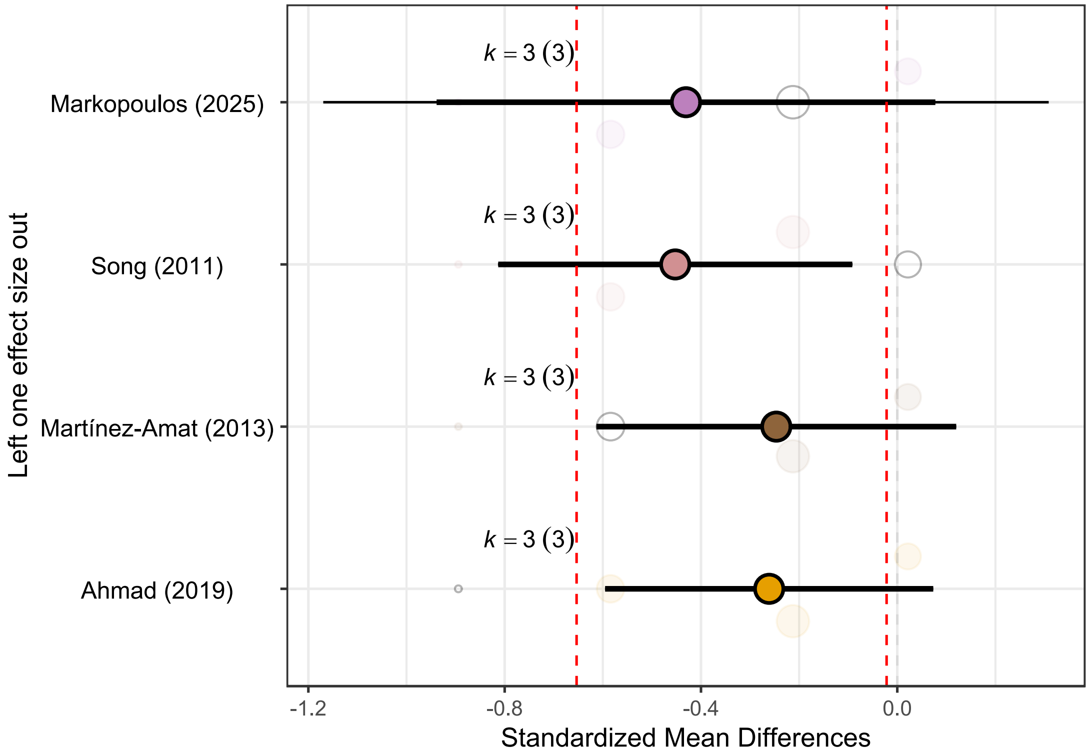

Fig A28. Leave-one-out sensitivity analysis for COP-EC.

Appendix G: Certainty of Evidence (GRADE Assessment)

Table A10. GRADE level of evidence for this study's findings.

Certainty assessment							№ of patients		Effect	Certainty
№ of studies	Study design	Risk of bias	Inconsistency	Indirectness	Imprecision	Other considerations	Sensorimotor-based interventions	Control conditions	Absolute (95% CI)	
**Dynamic Balance (assessed with: Timed Up and Go Test)**										
19	Randomised trials	Not serious	Serious[a]	Not serious	Not serious	Publication bias strongly suspected[b]	443	446	SMD **0.89 SD lower** (1.2 lower to 0.57 lower)	⊕⊕◯◯ Low[a,b]
**Static Balance (assessed with: Berg Balance Scale)**										
12	Randomised trials	Not serious	Not serious	Not serious	Not serious	None	305	310	SMD **0.94 SD higher** (0.7 higher to 1.18 higher)	⊕⊕⊕⊕ High
**Neuromuscular Control (COP-EO) (assessed with: Center of Pressure with eyes open)**										
7	Randomised trials	Not serious	Not serious	Not serious	Serious[c]	Publication bias strongly suspected[d]	133	130	SMD **0.66 SD lower** (0.97 lower to 0.35 lower)	⊕⊕◯◯ Low[c,d]
**Neuromuscular Control (COP-EC) (assessed with: Center of Pressure with eyes closed)**										
4	Randomised trials	Not serious	Not serious	Not serious	Serious[e]	None	79	82	SMD **0.34 SD lower** (0.65 lower to 0.02 lower)	⊕⊕⊕◯ Moderate[e]

CI: Confidence interval; **SMD:** Standardised mean difference

Explanations

a. Downgraded once due to inconsistency: high heterogeneity ($I^2 = 79.26\%$).
b. Downgraded once due to publication bias: significant bias detected (Egger's test p = 0.005) and asymmetric contour-enhanced funnel plots.
c. Downgraded once due to imprecision: the total sample size (N = 263) did not meet the optimal information size.
d. Downgraded once due to publication bias: significant bias detected (Egger's test p = 0.024) and asymmetric contour-enhanced funnel plots.
e. Downgraded once due to imprecision: the total sample size (N = 161) did not meet the optimal information size.
