# Supplementary material for: Dose-response relationships of sensorimotor-based interventions on balance performance in older adults: A systematic review and meta-regression analysis
Source: PLoS One. 2026 Jul 23;21(7):e0354522. doi: 10.1371/journal.pone.0354522 (PMC13395370; doi:10.1371/journal.pone.0354522)
Supplement: S2 Appendix — Detailed results of the risk of bias assessment using the ROB2 tool. (PDF) [file pone.0354522.s003.pdf]

Intention-to-treat

| Unique ID | Study ID | Experimental | Comparator | Outcome | Weight | D1 | D2 | D3 | D4 | D5 | Overall |
|---|---|---|---|---|---|---|---|---|---|---|---|
| Freire (2024) | Freire (2024) | ST | Conventional exercises | TUGT↑ | 1 | + | ! | + | + | + | ! |
| Jimenez-Mazuelas (2024) | Jimenez-Mazuelas (2024) | ST | Conventional therapy | TUGT↑ | 1 | + | + | + | + | + | + |
| Shabir (2021) | Shabir (2021) | ST | Conventional exercises | TUGT↑ | 1 | + | + | + | + | + | + |
| Sedighi Darijani (2024) | Sedighi Darijani (2024) | ST | No training program | TUGT↑ | 1 | + | + | + | + | + | + |
| Cetinkaya (2025) | Cetinkaya (2025) | ST | Dance therapy | BBS↑ | 1 | + | + | + | + | + | + |
| Niajalili (2026) | Niajalili (2026) | ST | Conventional therapy | TUGT↑ | 1 | + | + | + | + | + | + |
| Sadiq (2025) | Sadiq (2025) | ST | Conventional exercises | BBS↑ | 1 | + | + | + | ! | + | ! |
| Ahmad (2019) | Ahmad (2019) | ST | Conventional therapy | TUGT↑ COP↑ | 1 | ! | + | + | ! | ! | ! |
| Da Silva (2013) | Da Silva (2013) | ST | Conventional therapy | TUGT↑ BBS↑ | 1 | + | + | + | + | + | + |
| Morat (2019) | Morat (2019) | IG1:ST(UN) IG2:ST | No training program | TUGT↑ COP↑ | 1 | ! | ! | + | + | + | ! |
| DEMİR (2022) | DEMİR (2022) | PT | No training program | BBS↑ | 1 | + | + | + | + | + | + |
| Espejo-Antúnez (2020) | Espejo-Antúnez (2020) | PT | Conventional therapy | TUGT↑ | 1 | + | + | + | + | + | + |
| Esposito (2021) | Esposito (2021 | PT | No training program | BBS↑ | 1 | + | ! | + | - | ! | - |
| Martínez-Amat (2013) | Martínez-Amat (2013) | PT | No training program | COP↑ | 1 | ! | + | + | + | ! | ! |
| Martínez-López (2014) | Martínez-López (2014) | PT | Conventional exercises | BBS↑ | 1 | + | + | + | + | ! | ! |
| Song (2011) | Song (2011) | PT | Conventional therapy | TUGT↑ BBS↑ COP↑ | 1 | + | + | + | + | + | + |
| Teixeira (2010) | Teixeira (2010) | PT | Conventional therapy | TUGT↑ BBS↑ | 1 | + | + | + | + | + | + |
| Markopoulos (2025) | Markopoulos (2025) | PNF | Conventional exercises | TUGT↑ BBS↑ COP↑ | 1 | + | + | + | + | + | + |
| George (2025) | George (2025) | PNF | Conventional therapy | BBS↑ | 1 | + | + | + | + | + | + |
| Cellatoğlu (2025) | Cellatoğlu (2025) | PNF | Conventional exercises | TUGT↑ | 1 | + | + | + | + | + | + |
| Kajbafvala (2025) | Kajbafvala (2025) | PNF | Aerobic exercise | TUGT↑ BBS↑ | 1 | + | + | + | + | + | + |
| Mesquita (2015) | Mesquita (2015) | PNF | No training program | TUGT↑ BBS↑ COP↑ | 1 | + | + | + | + | + | + |
| Lamp (2023) | Lamp (2023) | IG1:PNF(RS) IG2:PNF(SR) | Conventional exercises | TUGT↑ | 1 | + | + | + | + | + | + |
| Kim (2015) | Kim (2015) | PNF | Swiss ball training | TUG↑ COP↑ | 1 | + | + | + | + | ! | ! |

+ Low risk

! Some concerns

- High risk

D1 Randomisation process

D2 Deviations from the intended interventions

D3 Missing outcome data

D4 Measurement of the outcome

D5 Selection of the reported result
